# Trimetallaborides as starting points for the syntheses of large metal-rich molecular borides and clusters†
†Electronic supplementary information (ESI) available: All synthetic, computational and crystallographic details. CCDC 1420470–1420473. For ESI and crystallographic data in CIF or other electronic format see DOI: 10.1039/c5sc03206g


**DOI:** 10.1039/c5sc03206g

**Published:** 2015-10-20

**Authors:** Holger Braunschweig, William C. Ewing, Sundargopal Ghosh, Thomas Kramer, James D. Mattock, Sebastian Östreicher, Alfredo Vargas, Christine Werner

**Affiliations:** a Institut für Anorganische Chemie , Julius-Maximilians-Universität Würzburg , Am Hubland , 97074 Würzburg , Germany . Email: h.braunschweig@mail.uni-wuerzburg.de; b Department of Chemistry , Indian Institute of Technology Madras , Chennai 600 036 , India; c Department of Chemistry , School of Life Sciences , University of Sussex , Brighton BN1 9QJ , Sussex , UK

## Abstract

Treatment of an anionic dimanganaborylene complex ([{Cp(CO)_2_Mn}_2_B]^–^) with coinage metal cations stabilized by a very weakly coordinating Lewis base (SMe_2_) led to the coordination of the incoming metal and subsequent displacement of dimethylsulfide in the formation of hexametalladiborides featuring planar four-membered M_2_B_2_ cores (M = Cu, Au) comparable to transition metal clusters constructed around four-membered rings composed solely of coinage metals. The analogies between compounds consisting of B_2_M_2_ units and M_4_ (M = Cu, Au) units speak to the often overlooked metalloid nature of boron. Treatment of one of these compounds (M = Cu) with a Lewis-basic metal fragment (Pt(PCy_3_)_2_) led to the formation of a tetrametallaboride featuring two manganese, one copper and one platinum atom, all bound to boron in a geometry not yet seen for this kind of compound. Computational examination suggests that this geometry is the result of d^10^–d^10^ dispersion interactions between the copper and platinum fragments.

## Abstract 2

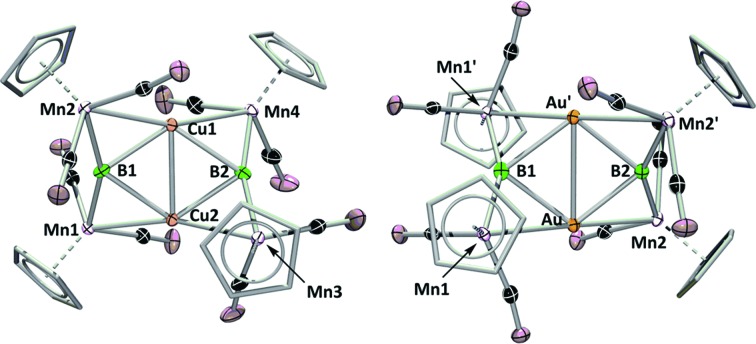
Treatment of an anionic dimanganaborylene complex with cationic coinage metal complexes led to the coordination of the incoming metal and displacement of dimethylsulfide in the formation of hexametalladiborides.

## Introduction

Dating from the early work of Lipscomb and fellow main group pioneers, the bonding arrangements behind the clustering of boron atoms into three dimensions has been methodically explored.^1^ Alongside these advances, the chemistry of related metallaboranes has briskly proceeded in the shadow of organometallic chemistry.^2^ Several reviews and articles have demonstrated that the progress made in the study of metal-rich metallaboranes is comparable to that coming from work on boron-rich, metal-containing cage clusters.^3^–^5^ To date, there has been no need to evaluate these systems in a comparative sense, as almost all known metallaboranes containing late transition metal fragments follow the same structural paradigm as polyhedral boranes.2a,3d,3e This fact is a clear measure of the success and utility of cluster-electron counting rules and the isolobal analogy linking boranes, metallaboranes, and metal clusters in a simple, conceptually pleasing fashion.^6^–^8^

The relationship between molecular clusters and solid state materials has drawn interest and engendered discussions.2*a* From early on in the study of transition metal clusters, it has been postulated that these groupings of metal atoms linked by metal–metal bonding arrangements were capable of simulating the surfaces of bulk materials, thereby providing an opportunity to study chemistry at material interfaces.^9^ Bulk transition metal borides have found uses as super-hard materials,^10^ superconductors,^11^ and magnetic materials,^12^ and the doping of bulk materials with boron is a commonly utilized strategy to augment electronic properties.^13^ It follows that the construction of polymetallic molecular structures featuring one or more boron atoms might provide a strategy for studying both the electronics and surface chemistries of metal borides, as well as effects induced by boron doping.

**Fig. 1:**
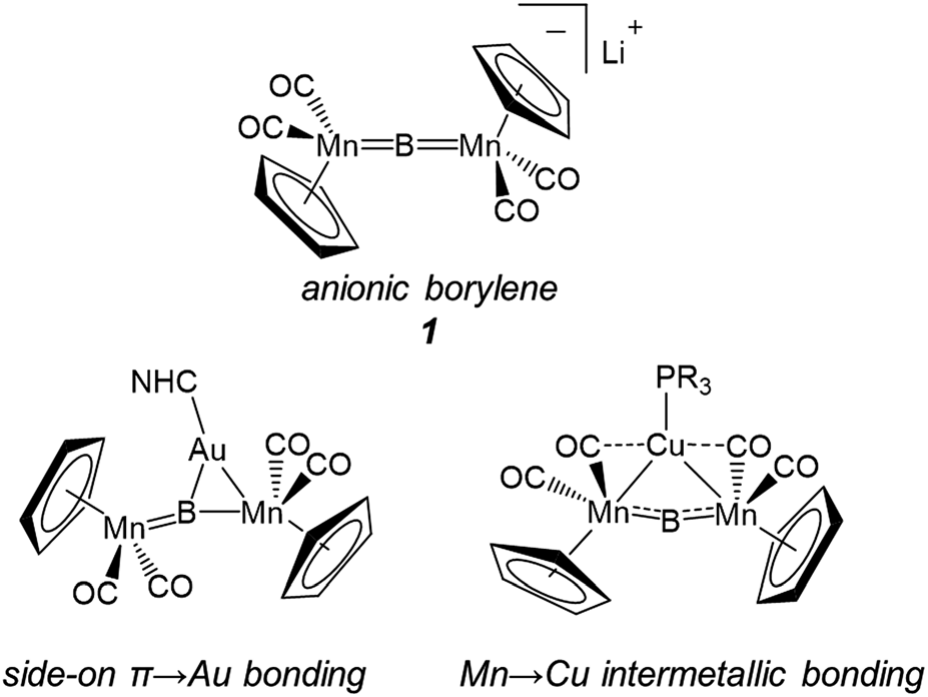
Complex **1** and examples of the two bonding motifs formed in reactions with base-stabilized cationic coinage metals.

For some time our group has been interested in developing methods for the systematic construction of such metal-rich molecular transition metal borides featuring three or more metals directly bonded to boron.^14^ In hopes of thoroughly understanding what we envision as an entry point into more metal-rich materials, we recently investigated the structural effects involved in changing both metals and ligands in a set of trimetallaboride complexes formed of the interactions of base-stabilized Lewis acidic coinage metal cations with an anionic dimanganaborylene ([{Cp(CO)_2_Mn}_2_B]^–^, **1**).^15^ In these compounds, the coinage metal is found complexed to the linear [Mn=B=Mn] unit of the borylene either in a position equidistant from the two Mn centers, in an arrangement held together by intermetallic bonds between the incoming metal and Mn, or spanning one of the B=Mn bonds, using the π-system as a side-on ligand with interactions roughly described by the Dewar–Chatt–Duncanson bonding model (Fig. 1).15*d*

Which of these bonding geometries a complex took, was found to depend on the combination of the metal's identity (Cu, Ag, or Au) and its stabilizing base. The magnitudes of both the σ-basicity and π-acidity of the three ligands studied, tricyclohexylphosphine (PCy_3_), 1,3-bis(4-tolyl)imidazol-2-ylidene (ITol), and 1-(2,6-diisopropylphenyl)-3,3,5,5-tetramethylpyrrolidin-2-ylidene (CAAC), had a direct influence on the bonding preferences of their accompanying coinage metals. Despite the observed differences, each of the three ligands explored in ref. 15d may be considered a strong σ-donor, limiting the overall range of the σ-acidity of the metal fragments studied. To explore the bonding of highly σ-acidic metal fragments, a weaker stabilizing ligand was required.

## Results and discussion

**Scheme 1:**
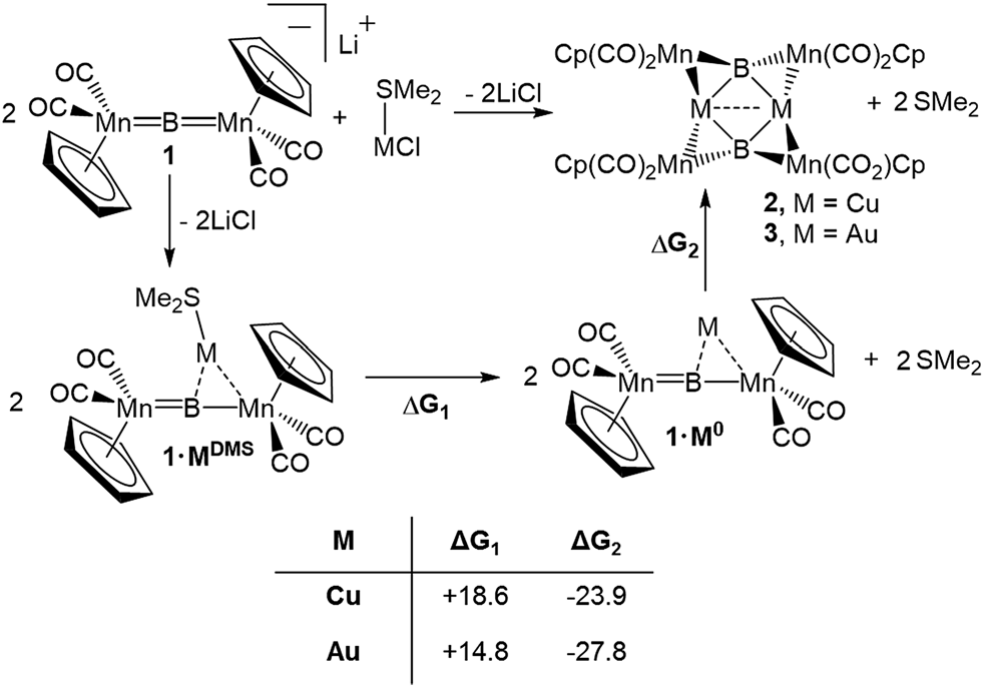
Reaction of **1** to **2** and **3** and the calculated energetics of the two postulated constitutive steps. For **2**, the values of Δ*G* were calculated in the gas phase at 298.15 K at the B3LYP/6-311 + G(d,p) level for all atoms. For **3**, the values of Δ*G* were calculated in the gas phase at 298.15 K and the B3LYP/6-311 + G(d,p) level for all small atoms and with the LANL2DZ pseudopotential for Mn and Au.

**Fig. 2:**
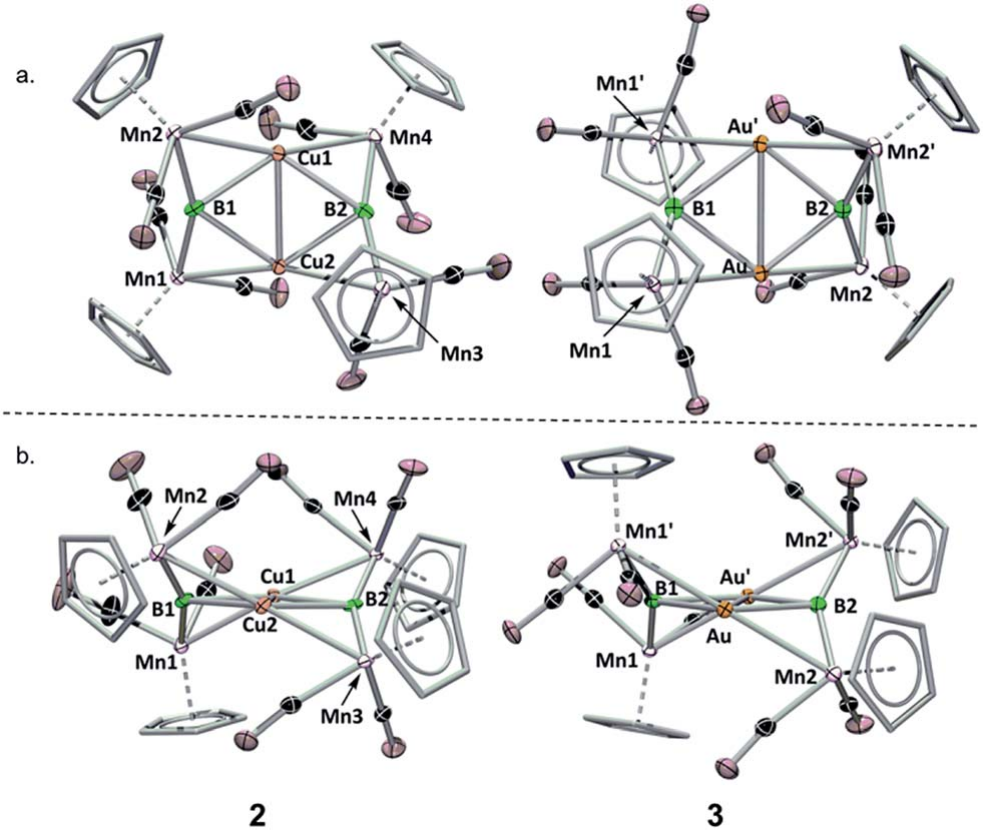
Crystallographically determined solid state structures of **2** and **3**. (a) View from above the M_2_B_2_ plane; (b) view along the M_2_B_2_ plane. A table of important bond lengths and angles is provided in the ESI (Table S2 and S3†).

To assess the effects of a highly σ-acidic metal stabilized by a weak σ-donor, we synthesized AuCl and CuCl stabilized by dimethyl sulfide (DMS) and reacted each with **1** (Scheme 1). In both cases, the reaction solution turned red concomitant with the formation of a white precipitate (presumably LiCl). The conversion of **1** was monitored by the disappearance of its ^11^B NMR resonance at 199 ppm, and the growth of a new peak at 208 ppm (in both reactions). As this chemical shift fell near the range established as normal for trimetallaborides (∼209–216 ppm),^15^ it was initially assumed that the reactions proceeded to yield similar compounds with the DMS ligand intact. However, X-ray analyses of painstakingly-grown single crystalline material did not show the expected trimetallaboride, but instead dimerization of two sulfide-free trimetallaborides in the formation of hexametalladiborides containing two coinage metals and four manganese centers (Scheme 1, Fig. 1) (Fig. 2).

**Fig. 3:**
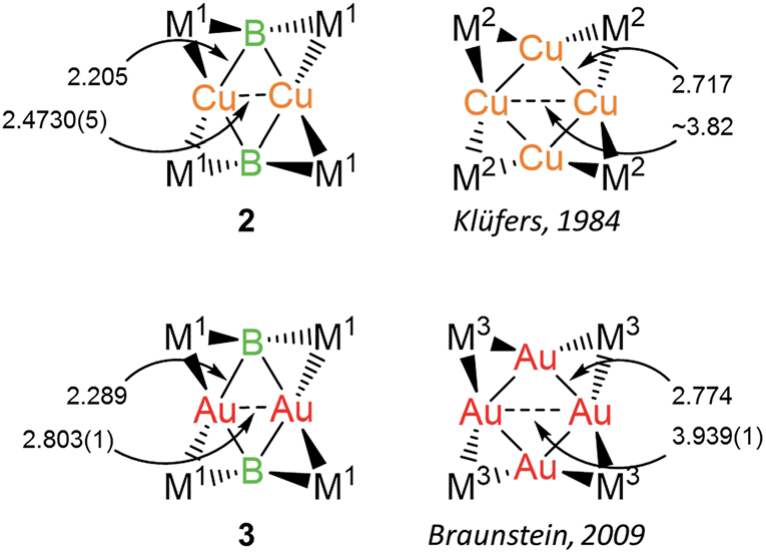
Comparison of **2** and **3** with compounds consisting of Cu_4_ and Au_4_ cores. Bond lengths are given in Å. M^1^ = Cp(CO)_2_Mn; M^2^ = (CO)_4_Co; M^3^ = Cp(CO)_2_Mo.

Both **2** and **3** consist of planar M_2_B_2_ units with [Cp(CO)_2_Mn] fragments bridging each M–B bond, alternating in positions above and below the M_2_B_2_ plane. This framework is reminiscent of a class of transition metal cluster complexes constructed around homometallic four-membered planar cores of naked coinage metal ions, with each M–M bond similarly bridged by a transition metal fragment.^16^**2** and **3** can be directly compared to two such compounds, the Cu_4_[(CO)_4_Co]_4_ and Au_4_[Cp(CO)_2_Mo]_4_ clusters reported by Klüfers and Braunstein, respectively (Fig. 3).16b,e The size difference between boron and either gold or copper can be expected to induce changes in the core, in this case distorting the central squares into diamond-shaped geometries with elongated B···B distances and short M···M distances. In Braunstein's Au compound, the cross-center Au···Au distances are 3.9391(11) and 3.9041(10) Å. The 2.8021(4) Å between the Au atoms of **3** is well shorter, instead falling in the range of the Au–Au edge-bonds, which measure between 2.7417(8) and 2.8030(9) Å. The Cu–Cu bond length in **2** (2.4730(5) Å) is substantially shorter than the edge Cu–Cu bonds found in Klüfer's complex (2.703(4)–2.731(4) Å), and even shorter than bonds comprising various triangular arrangements of Cu atoms (∼2.58–2.67 Å),14*a* which are comparatively more common.

The mimicry of the coinage metals by boron is interesting. Despite its position on the periodic table within the span occupied by elements commonly thought of as “metalloids”, boron has most often been treated solely as a non-metal.^17^ However, among those interested in metal–boron interactions, the similarities between boron and conventional metals (*i.e.*, open valence shells, Lewis acidity, lower electronegativity than hydrogen, *etc.*) have been noted, and comparisons have been drawn between the chemical behaviors of boron and metals, both in bulk materials and discrete molecules.^18^ Indeed, recent years have proven boron to be increasingly metal-like in many ways, such as in its propensity to coordinate and reductively couple multiple Lewis bases, including CO molecules.^19^ The existence of boron in homonuclear planar geometries^20^ and the fact that boron-rich planar clusters, such as the B_3_Fe_3_-core of a [(1,2,4-^*t*^Bu-C_5_H_2_)FeBH_2_]_3_ compound reported by Walter (albeit with bridging hydrogens),^21^ are already known speak to the element's versatility.

The syntheses of **2** and **3** are assumed to follow a pathway involving the initial formation of the triangular trimetallaboride complex (**1·M^DMS^**) followed by loss of DMS and dimerization (Scheme 1). Dative bonds between coinage metals and DMS are known to be very weak. Computational analysis indicates that the free energy of dissociation of DMS from **1·M^DMS^** is 7.4 kcal mol^–1^ in the case of Au and 9.8 kcal mol^–1^ for Cu (Scheme 1), leaving Au and Cu with empty coordination sites (**1·Cu^0^** and **1·Au^0^**). In contrast, the analogous release of an *N*-heterocyclic carbene ligand (IMe, 1,3-dimethylimidazol-2-ylidene) or simple phosphine (PMe_3_, trimethylphosphine) from a the Au complex requires 33.2 kcal mol^–1^ (IMe) and 20.7 kcal mol^–1^ (PMe_3_), and 30.3 kcal mol^–1^ (IMe) and 18.5 kcal mol^–1^ (PMe_3_) from the Cu complex. As the Δ*G* values for the dimerization of two liberated fragments in the formation of **2** and **3** are –23.9 and –27.8 kcal mol^–1^, respectively, the process of ligand-loss and subsequent dimerization is only downhill for the DMS-bound metals, explaining the fact that such dimerization has not been previously observed in reactions with either phosphine- or NHC-stabilized trimetallaborides. The treatment of **2** and **3** with PCy_3_ led to separation of each complex into two equivalents of **1·M^PCy3^**, in line with the calculated thermochemistry. The computed energies of each compound are compiled in Table S1.†

**Fig. 4:**
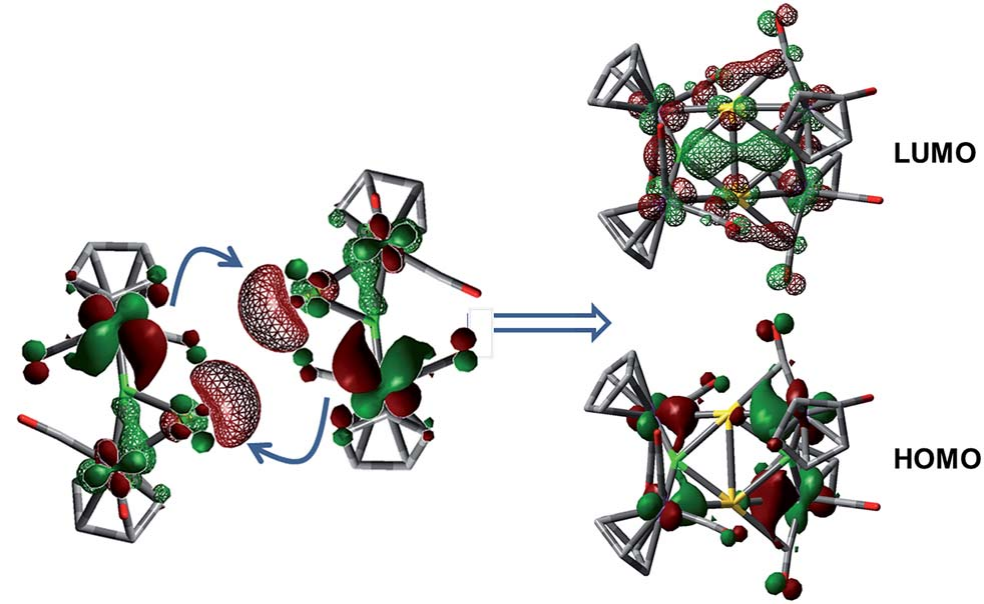
Depictions of the HOMO → LUMO interactions involved in the dimerization of **1·Au^0^** to **3**. Solid MO surfaces correspond to the HOMO, while thatched surfaces correspond to the LUMO. Structures were optimized with the B3LYP hybrid functional using the 6-311 + G(d,p) basis set for all light atoms and LANL2DZ for all metals.

The **1·M^0^** complexes are themselves stronger ligands for open coordination sites on coinage metal cations than DMS, but weaker ligands than NHCs or phosphines. Such reactivity, of course, requires them to be amphoteric in a Lewis sense. In the case of Au, these fragments are in the proper orientation for direct dimerization *via* simple HOMO/LUMO interactions (Fig. 4). The HOMO (solid lobes) is primarily comprised of a B–Mn π-bond, distorted toward the Mn atom unbound to Au. The LUMO (thatched lobes) is unsurprisingly situated predominantly at the open coordination site of the coinage metal. In the case of copper, the DFT optimized structure of **1·Cu^0^** shows the orientation of the copper atom directly above boron, seemingly blocking the approach route required for dimerization. However, the potential energy surface for the movement of the Cu atom out of the minimum toward one or the other Mn atom is rather shallow (Fig. S1†), allowing the Cu atom to easily move into an unsymmetrical position. This movement opens the required coordination site along the opposite B–Mn bond, exposing the Lewis basic segment of the HOMO required for donation to the acidic LUMO centered on the Cu atom.

**Fig. 5:**
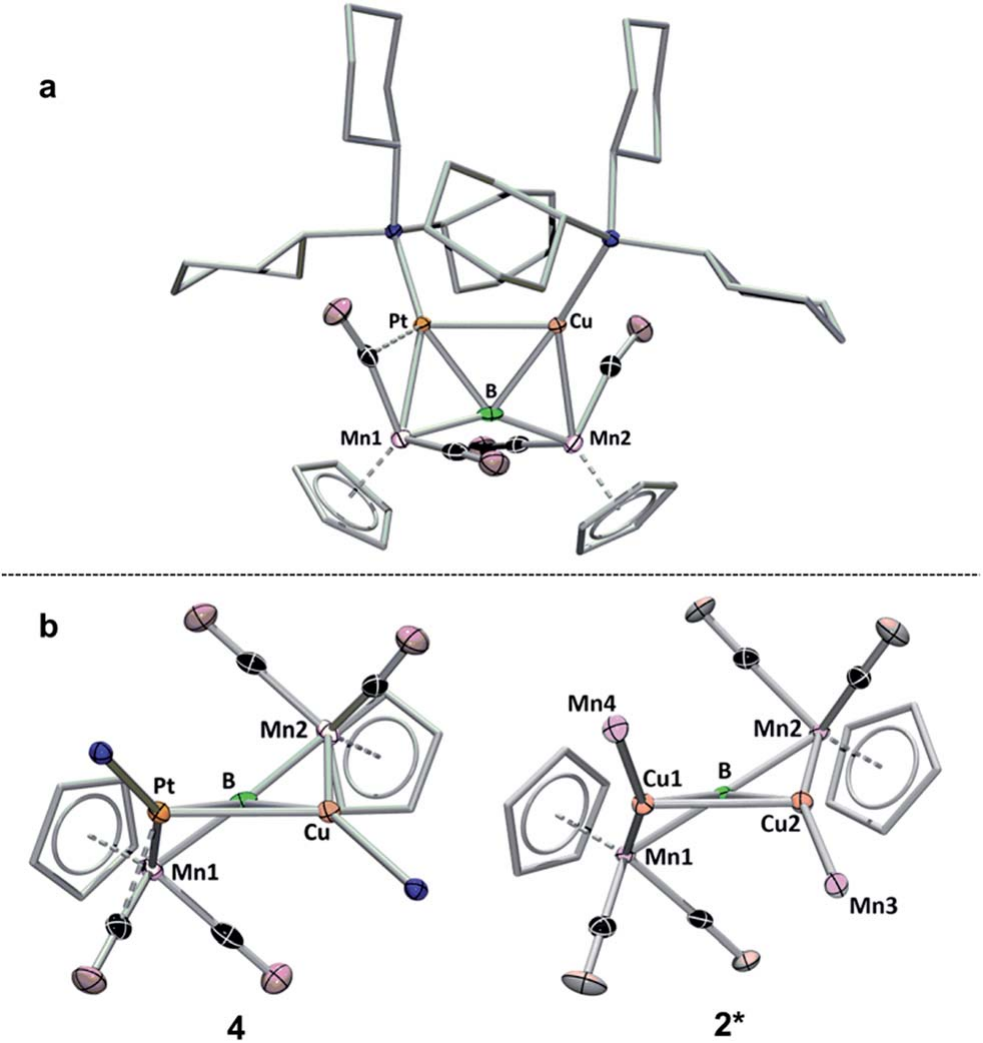
Two views of the crystallographically determined structure of **4**. (a) View from above the B–Pt–Cu plane; (b) comparison of **4** viewed along the B–Pt–Cu plane (with the cyclohexyl groups on the phosphines omitted) with a truncated version of **2** (2*) viewed along the Cu–B–Cu plane. A full table of the relevant bond lengths and angles is provided in Table S3.†

The easy separation of **2** and **3** into monomers by phosphine addition led us to treat **2** with the Lewis-basic transition metal complex Pt(PCy_3_)_2_. The addition of two equivalents of Pt(PCy_3_)_2_ to a toluene solution of **2** led to the formation of a new product with an ^11^B NMR peak at 215 ppm. Crystallographic isolation indeed showed the splitting of **2**, but instead of addition to the Lewis acidic Cu atom, Pt(PCy_3_)_2_ was found to have donated one phosphine to Cu and inserted along the adjacent Mn–B bond, immediately next to Cu, yielding a tetrametallaboride (**4**, eqn (1), Fig. 5a).
1

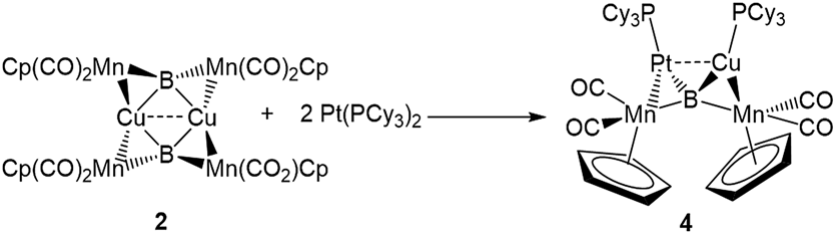




A view of compound **4** along the plane formed by the B, Cu and Pt atoms (Fig. 5b) shows the extent to which the product resembles **2**. An abbreviated structure 2* (the ligand architecture has been removed from two of the four Mn atoms) is displayed with the line-of-sight along the Cu–Cu–B plane. The above- and below-plane positions of Mn1 and Mn2 in **4** mimic the positions of Mn1 and Mn2 in 2*, while Mn3 and Mn4 in 2* have been replaced by PCy_3_ ligands in **4**. This observation lends support to the notion that B=Mn π-bonds, such as those in **1**, may act as σ-donating side-on ligands in their interactions with metals.15c,15d,^22^

**Fig. 6:**
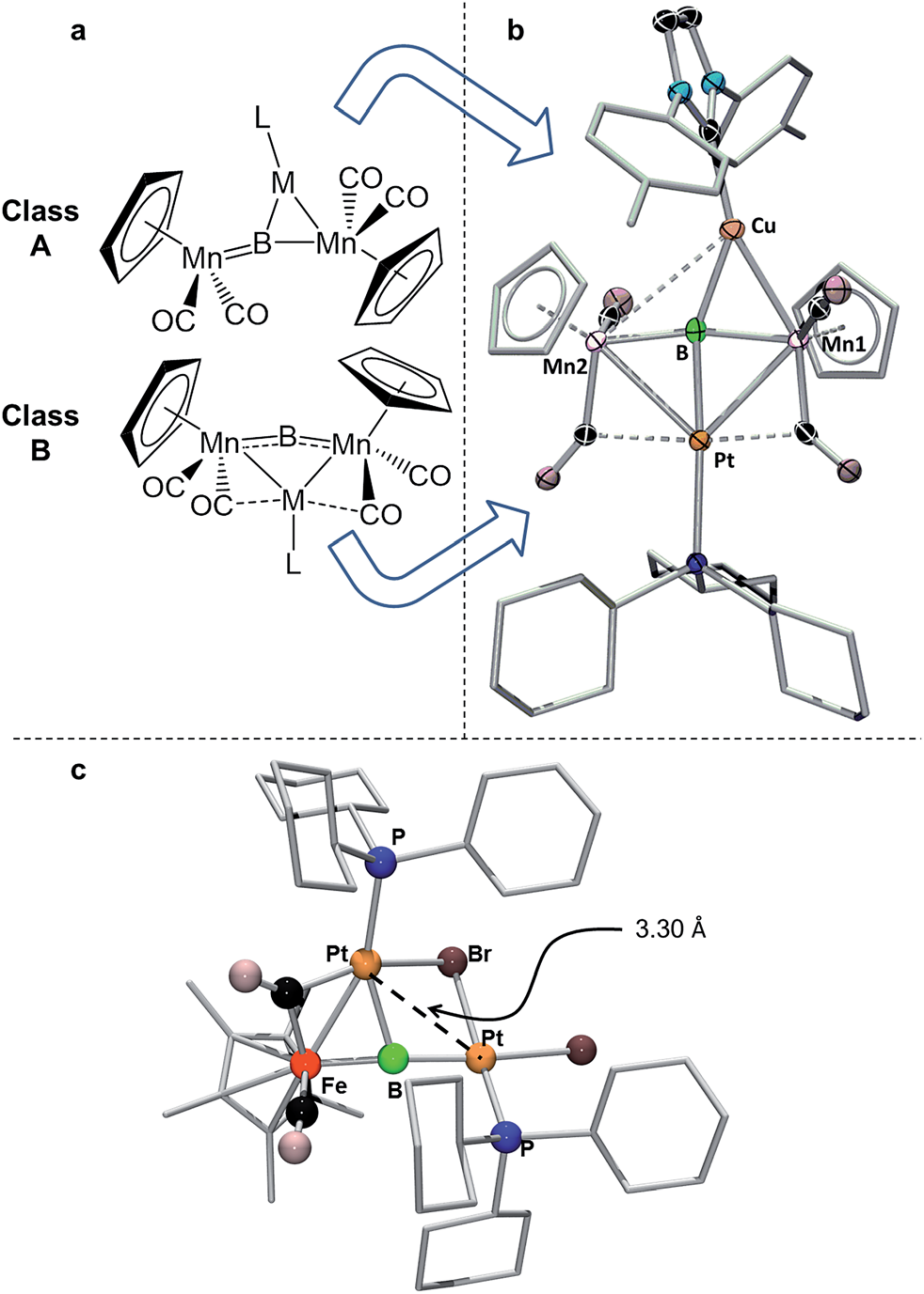
(a) Depiction of the two classes of bonding adopted by d^10^ metal fragments in conjunction with **1**; (b) the crystallographically determined solid state structure of **7**. Thermal ellipsoids indicate 50% probability, and have been omitted from the ligand architecture for clarity, as have all hydrogen atoms. A full list of relevant bond lengths and angles for **7** is provided in Table S4.† (c) Previously reported structure of the adduct of (PCy_3_)Pt and a ferraplatinaborylene.

This result was particularly surprising in light of the fact that a tetrametallaboride consisting of **1** coordinated to Pt(PCy_3_) and Au(ITol)^+^ has been reported, but in an altogether different geometry.14*a* This compound (**5**) was formed through the reaction of [{Pt(PCy_3_)}{Cp(CO)_2_Mn}_2_B]^–^ (**6**)14*a* with ITol–AuCl giving a tetrametallaboride with the boron in a distorted square planar geometry (eqn (2)). Still, the reaction of PCy_3_–CuCl and **6** again yielded **4** (eqn (3)). When the ligand on copper was changed for an NHC (ITol–CuCl) the same reaction (eqn (4)) led to the formation of a new product with an ^11^B NMR peak at 226 ppm, close to the shift reported for **6** (224 ppm). Single crystal X-ray analysis confirmed the distorted planar geometry of the boride, with the platinum fragment situated symmetrically between the Mn atoms, and the Cu bridging one of the two B–Mn bonds (**7**, Fig. 6b).
2

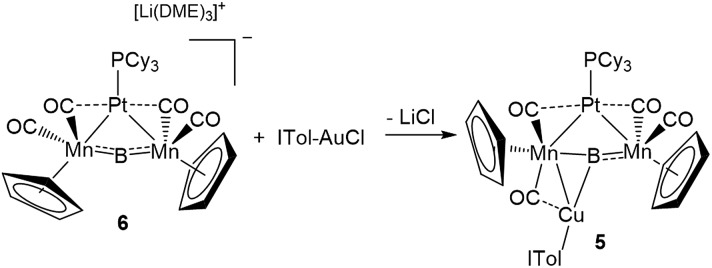



3

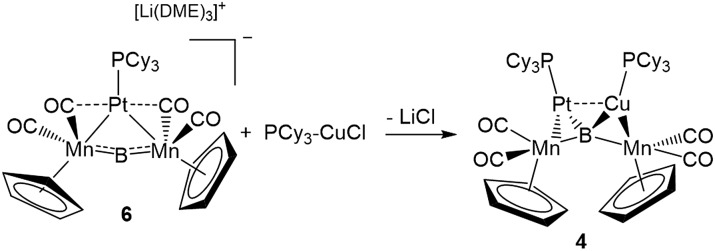



4

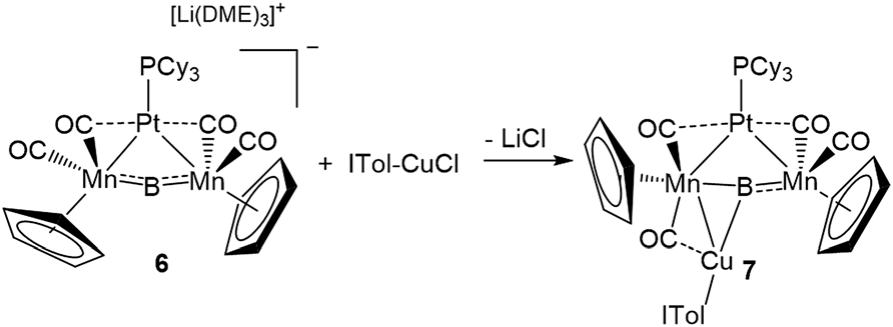




As mentioned, we recently described two different bonding motifs assumed by d^10^ coinage metal fragments in conjunction with **1**.15*d* The first of these is a side-on bonding arrangement, wherein an incoming coinage metal accepts donation from the B=Mn π-bond and participates in back bonding with a π* orbital (Fig. 6a, Class A). In such a bonding scheme, the [M–L] fragment bridges one of the two B–Mn bonds in **1**. In the second scenario, favored by metals that seek to maximize their coordination number (Fig. 6b, Class B), the metal takes a position immediately atop boron, distorting the [Mn–B–Mn] unit to minimize the bonding distance between the coinage metal and the Mn atoms on either side of the structure. The structure of **7** shows the presence of both bonding classes within the same compound. On one side of the [Mn–B–Mn] backbone the Cu(ITol)^+^ fragment bridges the B–Mn1 bond in an arrangement typical of Class A bonding, while on the other the Pt(PCy_3_) fragment adopts a Class B geometry, bending the [Mn–B–Mn] backbone (168.2(3)°) to maximize intermetallic bonding of both Mn1 and Mn2 with Pt. As was found typical of Class A bonding, the Cu-bridged Mn1–B bond (1.970(5) Å) was elongated with respect to the Mn2–B bond (1.907(5) Å). As typical for Class B bonding, the Mn1–Pt (2.707(1) Å) and Mn2–Pt (2.731(1) Å) bonds were similar, and both shorter than the sum of the covalent radii of Mn and Pt (2.75 Å) as determined by Cordero.^23^ Additionally, significant interactions are seen between Pt and the flanking CO ligands (Mn–C–O 147.6(4)°; Mn–C–O 140.0(3)°) as was found to be prevalent in all Class B bonding arrangements.

This description of the Pt(PCy_3_) fragment as Lewis acidic is at odds with previous descriptions of the behavior of this fragment in similar trimetallaborides. Calculations on a compound composed of a Pt(PCy_3_) fragment complexed to a dimetallaborylene backbone consisting of a boron atom between iron and platinum (Fig. 6c) described Pt(PCy_3_) as a Lewis base stabilizing an electrophilic boron.14*d* There are notable structural differences between the two cases. The bend in the borylene backbone, which minimizes the Mn–M distance in Class B bonding,15*d* is not present in the [Fe–B–Pt] backbone, which instead shows a slight bend in a perpendicular direction. Additionally, while the Mn–Pt bonds in **7** are both shorter than the sum of covalent radii, the Pt–Pt distance in the complex with the [Fe–B–Pt] backbone is roughly 3.3 Å, significantly longer than the 2.72 Å sum of covalent radii, indicating little metallophilic interaction between the incoming Pt(Cy_3_) fragment and the Pt in the borylene backbone. Subsequent reports from our group used this description of Pt(Cy_3_) as a base in conjunction with the [Mn–B–Mn] backbone;14*b* however, the geometrical features of this compound more closely resemble Class B bonding than those found in the computationally evaluated ferradiplatinaboride. It thus seems reasonable to assume that this versatile fragment is capable of either basic or acidic character, depending on its surrounding environment.

The comparison made in Fig. 5 describes **2–4** as consisting of conventional covalent bonding arrangements; however, the similarities of our materials to the cluster compounds described in Fig. 3 naturally led us to attempts at explaining the bonding using the Mingos fusion formalism.^7^ Within this framework, the total cluster valence electron (cve) counts for **2** and **3** are both 92. However, if it is assumed that each metal utilizes 9 atomic orbitals (AO), the cve is tabulated as 98. If instead the four lighter metals (Mn) use 9 AO and two heavier metals (Cu and Au) use 8 AO, the cve counts become 94. Cluster **4** is unique in that it possesses 60 cve; however, if the same approach is applied as for **2** and **3**, it yields 66 cve, in the case where all metals use 9 AO, and 62 when Mn uses 9 and Pt/Cu use 8 AO. The extra electrons are perhaps more localized on the metal centers and thus are not involved in skeletal bonding. It is common for heavier transition metals, *e.g.* Pt and Au, to form complexes with either 16 or 14 valence electrons,7*c* as reflected in the bonding of **2–4**.

The differences between **4** and **7** are perplexing. It is perhaps attractive to view the systems as examples of four-coordinate boron exiting in both distorted planar (**7**) and distorted tetrahedral (**4**) geometries; however, the distortions from ideal tetrahedral geometry in **4** are rather large, with angles as large as 148° and as small as 70°. Another possible explanation of the observed differences stems from the possibility of metallophilic interactions between the Cu and Pt in **4**, and a lack of these interactions in **7**. Closed shell, d^10^–d^10^ interactions involving these two nuclei are rare in comparison to examples involving gold;^24^ however, such a Pt(0)–Cu(i) interaction has been suggested as possible by a combined HF-DFT-MP2 study of simple model compounds.^25^ The Cu–Pt bond length in **4** (2.668(3) Å) is slightly less than the sum of covalent radii of the two metals (2.68 Å),^23^ suggesting an interaction. However, the planar geometry of **7**, featuring identical metals, seemingly contradicts this argument. Optimization of **4** using the OLYP functional failed to accurately reproduce the experimental Pt–Cu bond length, instead giving a much longer distance (2.83 Å). As it is well known that standard DFT methods do not adequately treat dispersion interactions,^26^ the optimization was repeated employing Grimme's semi-empirical dispersion correction,^27^ giving a structure with a much shorter Cu–Pt distance (2.60 Å, Table 1), which, while shorter than the experimental length, is in better agreement than the optimization without dispersion. The OLYP/TZP optimization of a compound wherein the Cu(PCy_3_)^+^ fragment in **4** was replaced by a Cu(IMe)^+^ fragment (**4^IMe^**)^28^ gave a structure with a Cu–Pt length of 2.75 Å. When Grimme's correction was applied to this optimization, the bond was again found to be shorter (2.62 Å, Table 1), but the difference between the corrected and uncorrected lengths was much smaller, indicating lesser effects from dispersion. Taken together, these data indicate a more prevalent influence of dispersion forces in **4** than a compound with an NHC-stabilized Cu atom.

**Table 1:** Comparison of the Cu–Pt bonds in **4** and **4^IMe^** with and without Grimme's dispersion correction. MBI = Mayer bond index; calculations were performed at the OLYP/TZP level, within the zeroth-order regular approximation (ZORA) formalism, with and without the D3 version of Grimme's dispersion with Becke–Johnson damping

	Cu–Pt (Å) without dispersion	Cu–Pt (Å) with dispersion	Cu–Pt (MBI) without dispersion	Cu–Pt (MBI) with dispersion
**4**	2.83	2.60	<0.01	0.43
**4^IMe^**	2.75	2.62	0.29	0.38

The influence of stabilizing ligands on the dispersion forces between closed-shell metal ions is a matter of open debate. In comparing model L–AuCl systems, Pyykk and coworkers found NHCs to promote the strongest dispersion forces in [L–AuCl]_2_ dimers as compared to a range of other ligands, including phosphines.^29^ While the dominant interaction in these dimers is the Au···Au interaction, van der Waals forces between metal ions and the ligand of the adjacent metal ion play an important role.^30^ In the computed [L–AuCl]_2_ dimers, these Au···L interactions were found to be the strongest when L = NHC, but only in conformations where the planes of the NHCs were parallel to one another. In other conformations, the stabilizing interaction is far smaller, falling below the computed strength of the Au···PH_3_ interaction in the [PH_3_–AuCl]_2_ dimer. Calculations at the MP2 level have indicated that in the case of a [PH_3_–CuCl]_2_ dimer the Cu···PH_3_ contribution to dispersion is the dominant term.^31^ Clearly, the optimal face-to-face NHC orientation cannot be achieved in the mixed ligand environment of **4^IMe^**, which perhaps limits the strength of the dispersion forces. Both the [Cu(PCy_3_)]^+^ and [Cu(ITol)]^+^ fragments are bulky, and from a strictly steric standpoint would favor inhabiting opposite sides of the molecule from the likewise bulky [Pt(PCy_3_)] fragment (the distorted square planar arrangement); however, the significant dispersion forces calculated for **4** may play the deciding role in the compound's observed geometry.

Perhaps even more interesting than the presence of strong dispersion forces in **4** is the seeming lack of them in **2** and **3**, especially when considering the rather short Cu···Cu and Au···Au distances. The optimized geometries for both of these compounds give M···M lengths of 2.48 and 2.82 Å for **2** and **3**, respectively, which are slightly greater than, but still in the range of, their experimental values (2.4730(5) Å, **2**; 2.803(1) Å, **3**). The application of Grimme's dispersion correction changed the optimized bond distances only slightly (2.45 Å, **2**; 2.85 Å, **3**). Though confirmation of this through *ab initio* methods is still needed, these findings suggest that the geometries of **2** and **3** are dictated by covalent bonding in the Mn_4_M_2_B_2_ core rather than by closed-shell dispersion interactions.

## Conclusions

The use of coinage metal ions with easily-displaced ligands provided a route to expanded metallaboride compounds containing six metal atoms. Systems such as these may find use in the future as mimics for the surfaces of boron-containing bulk materials. In these compounds the metalloid nature of boron is on display. These hexametalladiborides were split into tetrametallaborides through treatment with Pt(PCy_3_)_2_, giving structures that seem to rely on dispersion-type d^10^–d^10^ interactions for their shape.

Elucidation of the transition from boron-rich metallaboranes to metal-heavy transition metal borides may well lead to the discovery of bulk materials with many possible applications. Knowledge regarding the systematic syntheses of compounds within this continuum, such as the findings described here, are important tools in these efforts.

## Supplementary Material

Supplementary information

Crystal structure data
