# Supplementary material for: Trimetallaborides as starting points for the syntheses of large metal-rich molecular borides and clusters
Source: Chem Sci. 2015 Oct 20;7(1):109–16. doi: 10.1039/c5sc03206g (PMC5950574; doi:10.1039/c5sc03206g)
Supplement: Supplementary file 1 [file SC-007-C5SC03206G-s001.pdf]

Electronic Supplementary Material (ESI) for Chemical Science.
This journal is © The Royal Society of Chemistry 2015

Supporting Information for:

Trimetallaborides as Starting Points for the Syntheses of Large Metal-Rich Molecular Borides and Clusters

Holger Braunschweig, William C. Ewing, Thomas Kramer, Sundargopal Ghosh, Sebastian Östreicher, Alfredo Vargas, James Mattock and Christine Werner

h.braunschweig@uni-wuerzburg.de

Experimental

Materials: Li[{(CO)$_2$CpMn}$_2$B] (**1**),[1] dimethylsulfide gold and copper chlorides (DMS–AuCl, DMS–CuCl) and 1,3-(4-tolyl)imidazol-2-ylidene copper chloride (ITol–CuCl),[2] and Li[{(CO)$_2$CpMn}$_2$B{Pt(PCy$_3$)}] (**6**),[3] were prepared by literature methods.

Physical Methods: All manipulations were performed either under an atmosphere of dry argon or in vacuo using standard Schlenk line or glovebox techniques. All solvents (including DMS) were dried and degassed as according to literature methods, and stored under inert environments.[4] NMR spectra in solution were acquired on a Bruker Avance 400 (^{1}H: 400.1 MHz, ^{11}B{^{1}H}: 128.4 MHz, ^{13}C{^{1}H}: 100.6 MHz). ^{1}H NMR and ^{13}C NMR spectra were referenced to external TMS *via* residual protons of the solvent (^{1}H) or the solvent itself (^{13}C) and ^{11}B{^{1}H} NMR spectra were referenced to external $Et_2O \cdot BF_3$. IR spectra were acquired on a JASCO FT/IR-6200 type A spectrometer. Elemental analysis was performed on Vario MICRO Cube Elemental Analyzer.

Crystallographic Details: All structures were solved using direct methods, refined with the Shelx software package[5] and expanded using Fourier techniques. The *ShelXL* was interfaced with *ShelXLe* GUI for most of the refinement steps.[6] The pictures of molecules were prepared using *Pov-Ray* 3.6.2.[7] Additional details on refinement can be found in CIF files, which can be obtained from The Cambridge Crystallographic Data Centre *via* www.ccdc.cam.ac.uk/data_request/cif under CCDC 1420470 (**2**), CCDC 1420471 (**3**), CCDC 1420472 (**4**), and CCDC 1420473 (**7**).

For **2**:

The crystal data of **2** were collected on a Bruker X8-APEX II diffractometer with a CCD area detector and multi-layer mirror monochromated $Mo_{K\alpha}$ radiation. Hydrogen atoms were included in structure factors calculations. All hydrogen atoms were assigned to idealised geometric positions.

Crystal data for **2**: $C_{28}H_{20}B_2Cu_2Mn_4O_8$, $M_r = 852.90$, orange block, 0.26×0.19×0.12 mm^3, Monoclinic space group $P2_1/c$, $a = 12.6570(12)$ Å, $b = 17.3227(16)$ Å, $c = 13.6878(13)$ Å, $\beta = 91.065(4)°$, $V = 3000.6(5)$ Å^3, $Z = 4$, $\rho_{calcd} = 1.888$ g·cm^{-3}, $\mu = 3.072$ mm^{-1}, $F(000) = 1680$, $T = 100(2)$ K, $R_1 = 0.0396$, $wR^2 = 0.0780$, 6139 independent reflections [$2\theta \leq 52.74°$] and 397 parameters.

For **3**:

The crystal data of **3** were collected on a Bruker X8-Apex II diffractometer with a CCD area detector and multi-layer mirror monochromated $Mo_{K\alpha}$ radiation. All non-hydrogen atoms were refined anisotropically. Hydrogen atoms were included in structure factors calculations. All hydrogen atoms were assigned to idealised geometric positions.

Crystal data for **tsk100**: $C_{28}H_{20}Au_2B_2Mn_4O_8$, $M_r = 1119.75$, red block, 0.12×0.04×0.03 mm^3, Monoclinic space group $C2/c$, $a = 17.4662(13)$ Å, $b = 12.1607(9)$ Å, $c = 15.4013(18)$ Å, $\alpha = 119.822(2)°$, $V = 2838.1(4)$ Å^3, $Z = 4$, $\rho_{calcd} = 2.621$ g·cm^{-3}, $\mu = 12.077$ mm^{-1}, $F(000) = 2080$, $T = 100(2)$ K, $R_1 = 0.0195$, $wR^2 = 0.0409$, 3023 independent reflections [$2\theta \leq 53.52°$] and 200 parameters.

For **4**:

The crystal data of **4** were collected on a Bruker X8-Apex II diffractometer with a CCD area detector and multi-layer mirror monochromated $Mo_{K\alpha}$ radiation. All non-hydrogen atoms were refined anisotropically. Hydrogen atoms were included in structure factors calculations. All hydrogen atoms were assigned to idealised geometric positions.

The displacement parameters of the fragments $CuPCy_3$ and $PtPCy_3$ were constrained to the same value.

The Uii displacement parameters of the fragments $CuPCy_3$ and $PtPCy_3$ were restrained with ISOR keyword to approximate isotropic behavior.

Crystal data for **4**: $C_{50}H_{76}BCuMn_2O_4P_2Pt$, $M_r = 1182.37$, red block, 0.24×0.18×0.11 mm^3, Triclinic space group P-1, $a = 11.1740(8)$ Å, $b = 11.1760(8)$ Å, $c = 19.8602(14)$ Å, $\alpha = 83.931(2)°$, $\beta = 83.831(2)°$, $\gamma = 75.995(2)°$, $V = 2384.3(3)$ Å^3, $Z = 2$, $\rho_{calcd} = 1.647$ g·cm^{-3}, $\mu = 3.993$ mm^{-1}, $F(000) = 1200$, $T = 100(2)$ K, $R_1 = 0.0680$, $wR^2 = 0.0966$, 9725 independent reflections [$2\theta \leq 52.74°$] and 491 parameters.

For **7**:

The crystal data of **7** were collected on a Bruker X8APEX diffractometer with a CCD area detector and multi-layer mirror monochromated $Mo_{K\alpha}$ radiation. All non-hydrogen atoms were refined anisotropically. Hydrogen atoms were included in structure factors calculations. All hydrogen atoms were assigned to idealised geometric positions.

Crystal data for **7**: $C_{70}H_{83}BCuMn_2N_2O_4PPt$, $M_r = 1426.67$, red block, 0.21×0.15×0.08 mm^3, Triclinic space group P-1, $a = 12.516(2)$ Å, $b = 13.405(3)$ Å, $c = 19.099(4)$ Å, $\alpha = 100.262(5)°$, $\beta = 97.963(5)°$, $\gamma = 95.107(5)°$, $V = 3101.3(10)$ Å^3, $Z = 2$, $\rho_{calcd} = 1.528$ g·cm^{-3}, $\mu = 3.061$ mm^{-1}, $F(000) = 1452$, $T = 100(2)$ K, $R_1 = 0.0642$, $wR^2 = 0.0913$, 13026 independent reflections [$2\theta \leq 53.86°$] and 753 parameters.

Computational Details:

For the investigation of potential closed shell interactions, geometry optimization was carried out using the Amsterdam Density Functional (ADF)[8] program at the OLYP/TZP.[9] All calculations were conducted within the zeroth-order regular approximation (ZORA) formalism.[10] To obtain

the singlet state, spin-restricted calculations were performed constraining the projection of the total electronic spin along a reference axis to 0. Frequency calculations were conducted to determine if each stationary point corresponds to a minimum. Reported bond orders are of the Mayer bond order type[11] and atomic charges were determined according to the Hirshfeld charge analysis.[12] Dispersion was included via the addition of the D3 version of Grimme's dispersion with Becke-Johnson damping.[13] The Jmol[14] program and the Graphical User Interface (ADF-GUI) – a part of the ADF package, were used for visualization purposes.

For the reported thermochemistry, calculations were performed in the Gaussian 09 software suite.[15] For compound **2** and its related thermochemistry, optimizations were carried out at the B3LYP/6-311+G(d,p) level of theory for all atoms and checked via frequency calculation to ensure the stationary point was a true minimum with no imaginary frequencies. The calculated electronic and free energies at 298.15 K are given in Table 1. For compound **3**, small atoms were calculated at the B3LYP/6-311+G(d,p) level, while Au and Mn were treated at with the LANL2DZ pseudopotential.

Synthetic Details

$\{[(\eta_5\text{-}C_5H_5)_2(CO)_4Mn_2]BCu\}_2$ (**2**): DMS–CuCl (5.0 mg, 0.031 mmol) was added to a solution of **1** (20 mg, 0.031 mmol) in toluene (1 mL) and stirred for 0.5 h at room temperature. The color of the reaction mixture changed from slightly yellow to red and a colorless precipitate formed. After a complete conversion, indicated by the emergence of a ^{11}B NMR signal at 208 ppm, the solution was filtered and stored at –35 °C. Slow crystallization led to the isolation of red crystals of **2** suitable for X-ray diffraction. For (**2**): 12.1 mg (0.015 mmol, 46%) ^{1}H NMR (C_6D_6,

400.1 MHz): δ = 4.58 (s, 10H, C_5H_5). $^{11}B\{^1H\}$ NMR (C_6D_6, 128.4 MHz): δ = 208. $^{13}C\{^1H\}$ NMR (C_6D_6, 100.6 MHz): δ = 225.7 (CO), 84.3 (s, C_5H_5). FT-IR (hexane): 1957 (br), 1942 (br), 1907 (br), 1878 (br), 1863 (br) cm^{-1}. Elemental analysis: calc'd (%): C: 39.43, H: 2.36; found (%) C: 40.22 H: 2.80.

$\{[(\eta_5\text{-}C_5H_5)_2(CO)_4Mn_2]BAu\}_2$ (3): DMS–AuCl (4.6 mg, 0.016 mmol) was added to a solution of **1** (10 mg, 0.016 mmol) in toluene (1 mL) and stirred for 0.5 h at room temperature. The color of the reaction mixture changed from slightly yellow to red and a precipitate was formed. After a complete conversion in the ^{11}B NMR spectrum to a low-field signal at 208 ppm was observed, the solution was filtrated and stored at –35 °C. Slow crystallization led to the isolation of red crystals of **3** suitable for X-ray diffraction. For **3**: 3.2 mg (0.003 mmol, 17%). ^1H NMR (C_6D_6, 400.1 MHz): δ = 4.85 (s, 10H, C_5H_5). $^{11}B\{^1H\}$ NMR (C_6D_6, 128.4 MHz): δ = 208. $^{13}C\{1H\}$ NMR (C_6D_6, 100.6 MHz): δ = 232.2 (CO), 83.9 (s, C_5H_5). FT-IR (hexane): 1986 (br), 1945 (br), 1927 (br), 1913 (br), 1878 (br) cm^{-1}. Elemental analysis: calc'd (%): C: 30.03, H: 1.80; found (%) C: 31.05 H: 1.75.

$\{[(\eta_5\text{-}C_5H_5)_2(CO)_4Mn_2B][CuPCy_3][PtPCy_3]\}$ (4): $Pt(PCy_3)_2$ (21.3 mg, 0.028 mmol) was added to a solution of (**2**) (12 mg, 0.014 mmol) in toluene (1 mL) and stirred for 4 at 60 °C. The color of the reaction mixture remained red, but in the ^{11}B NMR spectrum a low-field shift to 215 ppm was observed. The solution was concentrated and stored at –35 °C. After 4 weeks orange crystals of **4** suitable for X-ray diffraction were obtained. For **4:** 10.2 mg (0.009 mmol, 31%). ^1H NMR (C_6D_6, 400.1 MHz): δ = 4.75 (s, 10H, C_5H_5), 1.23 – 2.41 (br, 66H, PCy_3). $^{11}B\{^1H\}$ NMR (C_6D_6, 128.4 MHz): δ = 215. $^{13}C\{^1H\}$ NMR (C_6D_6, 100.6 MHz): δ = 236.9 (CO), 82.3 (s, C_5H_5), 25.0 –

34.9 (br, PCy_3). $^{31}P\{^1H\}$ NMR (C_6D_6, 162.0 MHz): δ = 63.9 (s, Pt*P*Cy_3, $^1J_{P\text{-}Pt}$ = 4421 Hz), 13.4 (s, Cu*P*Cy_3). FT-IR (hexane): 1946 (br), 1913 (br), 1873 (br), 1849 (br), 1793 (br), 1768 (br) cm^{-1}. Elemental analysis: calc'd (%): C: 50.79, H: 6.47; found (%) C: 51.06 H: 6.75.

Alternative route to **4**: PCy_3–CuCl (24.0 mg, 0.047 mmol) was added to a solution of **6** (30 mg, 0.047 mmol) in toluene (1 mL) and stirred for 0.5 h at room temperature. The color of the reaction mixture turned yellow and a white precipitate formed. In the ^{11}B NMR spectrum a low-field shift to 216 ppm was observed. The solution was filtered, concentrated and stored at -35 °C. After 12 h yellow crystals of **4** were obtained. Yield: 31 mg, 0.037 mmol, 79%.

(η_5-C_5H_5)$_2$(CO)$_4$$Mn_2$B(Pt$PCy_3$)(CuITol) (7): ITol–CuCl (3.71 mg, 0.010 mmol) was added to a solution of **6** (10 mg, 0.010 mmol) in toluene (1 mL) and stirred for 2 h at room temperature. The color of the reaction mixture turned red as a white precipitate formed. A downfield shift of the ^{11}B NMR resonance to 226 ppm was observed. The solution was filtered, concentrated and stored at –35 °C. After four weeks, orange crystals of **7** suitable for X-ray diffraction were obtained. For **7**: 7.4 mg (0.006 mmol, 61%). ^1H NMR (C_6D_6, 400.1 MHz): δ = 7.65 (d, 4H, *Tol*), 7.03 (t, 4H, *Tol*), 6.47 (s, 2H, *NHC*), 4.38 (s, 10H, C_5H_5), 2.74 (m, 3H, P*Cy3*), 2.18 (m, 6H, P*Cy3*), 1.96 (s, 6H, *Tol*), 1.77 (m, 6H, P*Cy3*), 1.63 (m, 6H, P*Cy3*), 1.36 (m, 6H, P*Cy3*), 1.20 (m, 6H, P*Cy3*). $^{11}B\{^1H\}$ NMR (C_6D_6, 128.4 MHz): δ = 226. $^{13}C\{1H\}$ NMR (C_6D_6, 100.6 MHz): δ = 214.3 (*C*O), 139.4 (*Tol*), 138.2 (*Tol*), 130.8 (*Tol*), 125.0 (*Tol*), 121.5 NHC), 82.0 (s, C_5H_5), 34.3 (P*Cy3*), 30.7 (P*Cy3*), 28.3 (P*Cy3*), 27.2 (P*Cy3*), 20.9 (*Tol*). $^{31}P\{^1H\}$ NMR (C_6D_6, 162.0 MHz): δ = 55.3 (s, Pt*P*Cy_3, $^1J_{P\text{-}Pt}$ = 4269 Hz). FT-IR (hexane): 1948 (br), 1909 (br), 1859 (br), 1766 (br) cm^{-1}. Elemental analysis: calc'd (%): C: 54.14, H: 5.44, N: 2.25; found (%) C: 54.29 H: 5.53, N: 2.13.

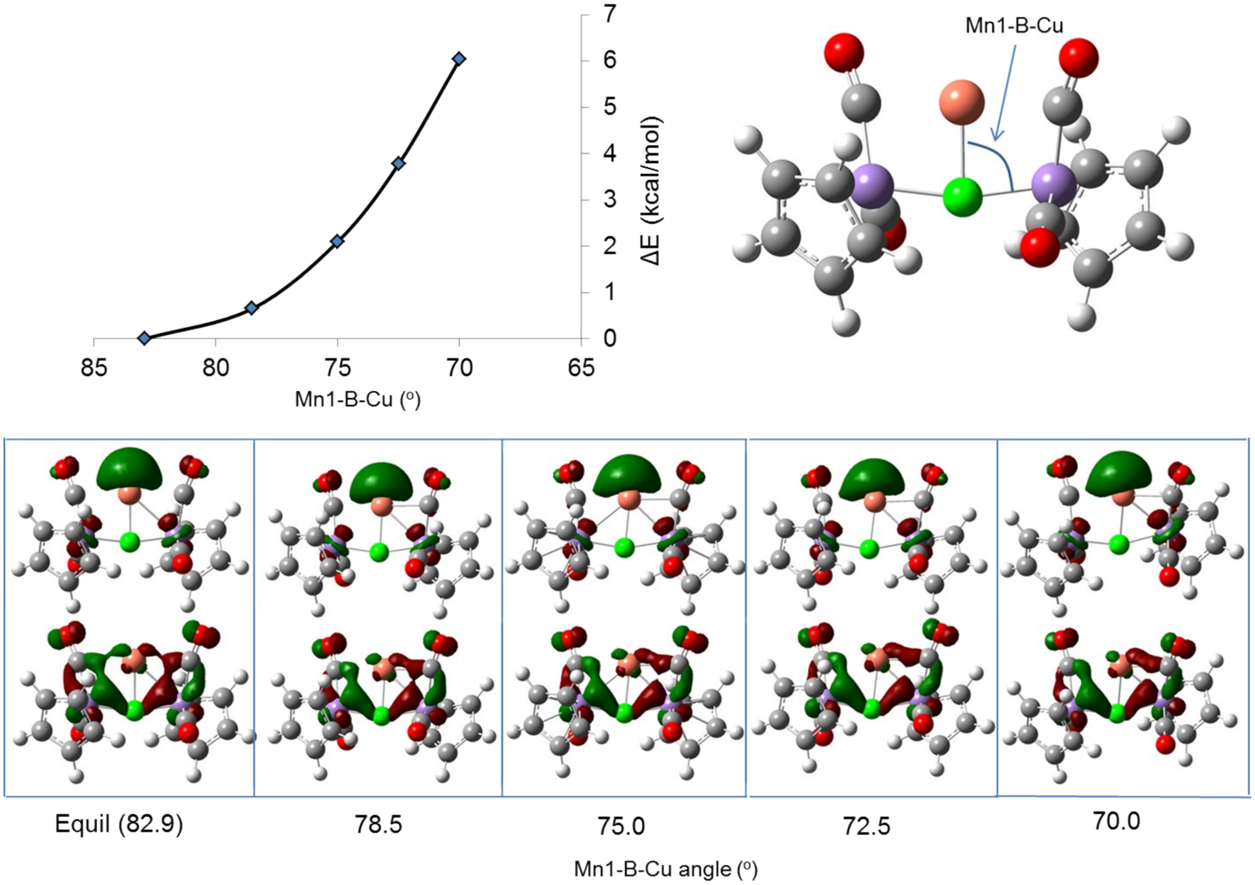

Figure S1. Evaluation of the potential energy changes involved in the movement of the Cu ion in 2a from a centrosymmetric position to a position closer to one of the two Mn atoms. Calculations were carried out at the B3LYP/6-311+G** level for all atoms by fixing the Mn1-B-Cu angle and optimizing around this coordinate. In the Kohn-Sham molecular orbital depictions, the top figure is the LUMO for the partially-optimized structures, and the lower figure is the HOMO. The values of E used for the determination of ΔE are not ZPE-corrected.

Table 1. Energies calculated for the reported thermochemical data. Energies are in Hartrees and calculated in the gas phase at 298.15 K. Optimizations and frequencies calculations were carried out using the B3LYP functional. For copper-containing compounds the 6-311+G(d,p) basis set was used for all atoms. For gold containing compounds the 6-311+G(d,p) basis set was used for all non-metals and the LANL2DZ pseudopotential was used for Mn and Au.

Compound	E	G
**1·Cu^{0}**	-4807.864936	-4807.918751
**1·Au^{0}**	-1208.65455	-1208.710171
**2**	-9615.79298	-9615.875567
**3**	-2417.380441	-2417.380441
**1·Cu^{DMS}**	-5285.889168	-5285.952871
**1·Au^{DMS}**	-1686.676566	-1686.740467
**1·Cu^{PMe3}**	-5268.963001	-5268.029736
**1·Au^{PMe3}**	-1669.758054	-1669.824634
**1·Cu^{IMe}**	-5112.685243	-5112.752845
**1·Au^{IMe}**	-1513.481545	-1513.548901
**DMS**	-477.991357	-478.018459
**PMe$_3$**	-461.052417	-461.081485
**IMe**	-304.754655	-304.785764

Table 2. Relevant bond lengths and angles for **2**.

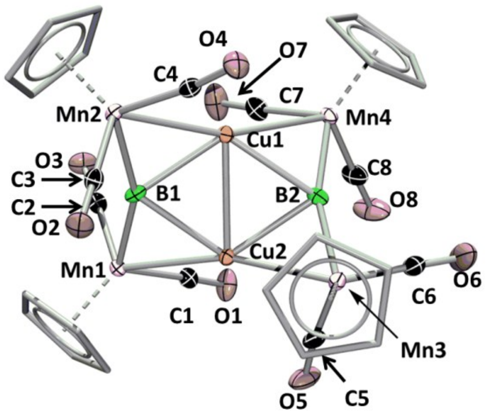

bond	length [Å]		atoms	angle (°)
B1-Mn1	1.960(3)		B2-Cu1-B1	111.13(12)
B1-Mn2	1.970(3)		Cu1-B1-Cu2	67.96(10)
B1-Cu1	2.186(3)		B1-Cu2-B2	112.40(12)
B1-Cu2	2.238(3)		Cu2-B2-Cu1	68.43(10)
B2-Mn3	1.975(3)		Mn1-B1-Mn2	161.95(19)
B2-Mn4	1.962(3)		Mn3-B2-Mn4	157.05(19)
B2-Cu1	2.240(3)		Mn2-Cu1-Mn4	162.90(2)
B2-Cu2	2.156(3)		Mn1-Cu2-Mn3	161.23(2)
Cu1-Cu2	2.473(1)			
Cu1-Mn2	2.458(1)			
Cu1-Mn4	2.464(1)			
Cu2-Mn1	2.440(1)			
Cu2-Mn3	2.462(1)		Mn1-C1-O1	173.0(3)
Cu1-C4	2.430(3)		Mn1-C2-O2	177.7(3)
Cu1-C7	2.264(3)		Mn2-C3-O3	178.3(3)
Cu2-C1	2.315(3)		Mn2-C4-O4	174.1(3)
Cu2-C5	2.281(3)		Mn3-C5-O5	171.0(2)
			Mn3-C6-O6	178.7(3)
C1-O1	1.168(4)		Mn4-C7-O7	172.8(2)
C2-O2	1.165(4)		Mn4-C8-O8	177.7(3)
C3-O3	1.157(4)			
C4-O4	1.166(3)			
Mn1-C1	1.804(3)			
Mn1-C2	1.780(3)			
Mn2-C3	1.778(3)			
Mn2-C4	1.797(3)			

C5-O5	1.172(3)			
C6-O6	1.162(3)			
C7-O7	1.159(4)			
C8-O8	1.167(3)			
Mn3-C5	1.797(3)			
Mn3-C6	1.778(3)			
Mn4-C7	1.817(3)			
Mn4-C8	1.775(3)			

Table 3. Relevant bond lengths and angles for **3**.

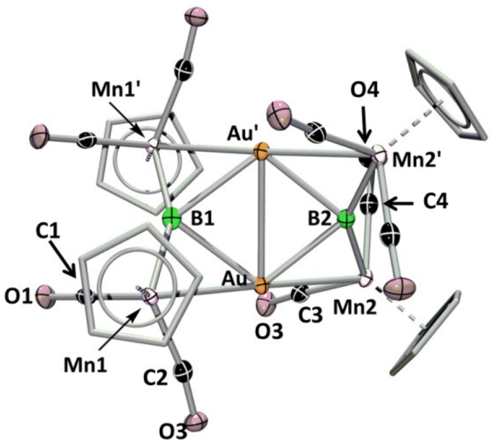

bond	length [Å]		atoms	angle (°)
B1-Mn1	1.983(2)		B2-Au-B1	104.48(12)
B1-Mn1'	1.983(2)		Au-B1-Au'	74.05(16)
B1-Au	2.327(4)		B1-Au'-B2	104.48(12)
B1-Au'	2.327(4)		Au'-B2-Au	76.99(17)
B2-Mn2	1.991(2)		Mn1-B1-Mn1'	155.9(3)
B2-Mn2'	1.991(2)		Mn2-B2-Mn2'	148.8(3)
B2-Au	2.251(4)		Mn1-Au-Mn2	178.47(2)
B2-Au'	2.251(4)		Mn1'-Au'-Mn2'	178.47(2)
Au-Au'	2.803(1)			
Au-Mn1	2.614(1)			
Au-Mn2	2.631(1)			
			Mn1-C1-O1	178.4(3)

			Mn1-C2-O2	169.7(3)
C1-O1	1.154(4)		Mn2-C3-O3	170.9(3)
C2-O2	1.156(4)		Mn2-C4-O4	177.8(3)
C3-O3	1.158(4)			
C4-O4	1.153(4)			
Mn1-C1	1.772(4)			
Mn1-C2	1.830(4)			
Mn2-C3	1.794(4)			
Mn2-C4	1.769(4)			

Table 4. Relevant bond lengths and angles in **4**.

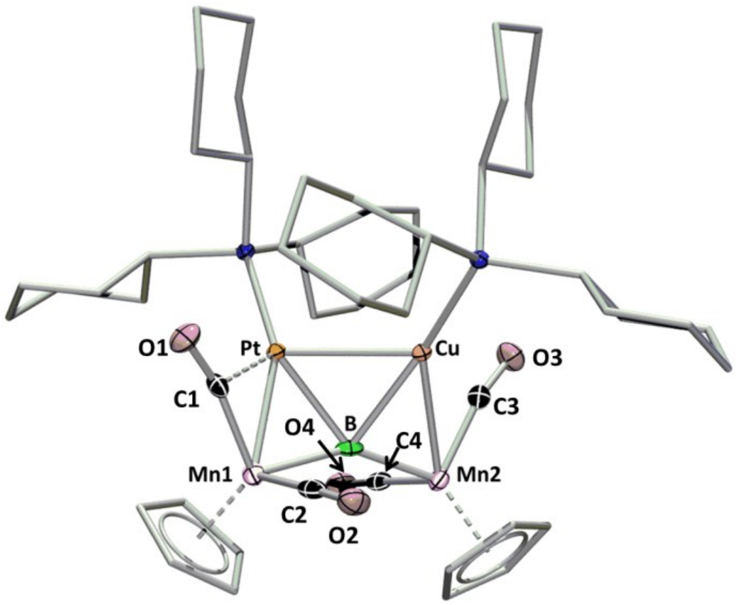

bond	length [Å]		atoms	angle (°)
B1-Mn1	1.970(6)		Mn1-B1-Mn2	149.1(3)
B1-Mn2	1.970(6)		Cu1-B1-Pt1	75.19(18)
B1-Cu1	2.176(6)		Mn1-B1-Pt1	72.02(18)
B1-Pt1	2.196(5)		Mn1-B1-Cu1	130.1(3)
Cu1-Mn1	2.623(3)		Mn2-B1-Pt1	78.3(2)
Pt1-Mn1	2.456(2)		Mn2-B1-Cu1	135.2(3)
Pt1-Cu1	2.668(3)			
			Mn1-C1-O1	164.7(4)
C1-O1	1.174(5)		Mn1-C2-O2	178.1(4)
C2-O2	1.176(6)		Mn2-C3-O3	164.6(4)
C3-O3	1.159(5)		Mn2-C4-O4	177.8(4)
C4-O4	1.170(6)		Pt1-C1-O1	117.8(4)
Mn1-C1	1.815(5)		Cu1-C3-O3	116.7(4)
Mn1-C2	1.756(6)			
Mn2-C3	1.824(5)			
Mn2-C4	1.756(5)			
			B1-Cu1-P2	153.1(3)
Pt1-C1	2.099(5)		Mn2-Cu1-P2	145.0(2)
Cu1-C3	2.275(5)		B1-Pt1-P1	149.2(2)
Pt1-P1	2.263(7)		Mn1-Pt1-P1	155.3(2)

Cu1-P2	2.259(9)			

Table 5. Relevant bond lengths and angles in **7**.

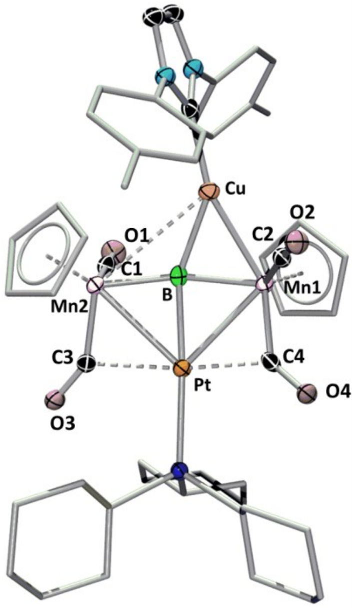

bond	length [Å]		atoms	angle (°)
B1-Mn1	1.907(5)		Mn1-B1-Mn2	168.2(3)
B1-Mn2	1.970(5)		Cu1-B1-Pt1	155.1(3)
B1-Cu1	2.055(5)		Mn1-B1-Pt1	85.6(2)
B1-Pt1	2.106(5)		Mn1-B1-Cu1	115.2(3)
Cu1-Mn1	3.347(1)		Mn2-B1-Pt1	83.14(19)
Cu1-Mn2	2.494(1)		Mn2-B1-Cu1	76.52(17)
Pt1-Mn1	2.707(1)			
Pt1-Mn2	2.731(1)			
C1-O1	1.176(5)		Mn1-C1-O1	178.8(4)
C2-O2	1.169(6)		Mn1-C3-O3	147.6(4)
C3-O3	1.173(5)		Mn2-C2-O2	176.1(4)
C4-O4	1.186(5)		Mn2-C4-O4	140.0(3)
Mn1-C1	1.747(5)		Pt1-C3-O3	129.5(3)
Mn1-C3	1.853(5)		Pt1-C4-O4	134.5(3)
Mn2-C2	1.769(5)			
Mn2-C4	1.933(5)			
Pt1-C3	2.253(4)		B1-Cu1-C	147.57(19)
Pt1-C4	2.059(4)		Mn2-Cu1-C	161.21(13)
Pt1-P	2.338(2)		B1-Pt1-P	174.51(14)
Cu1-C	1.920(5)			

Table S6. Coordinated for the optimized structures in the presence and absence of dispersion corrections. For the methods employed see the **Computational Details**. Columns are multi-page.

[{(CO)$_2$CpMn}$_2$BAu]$_2$ without dispersion

Au	6.919333	10.05712	1.93568
Au	6.716622	10.05717	4.745432
O	6.37159	5.481425	1.325237
O	7.594752	8.653916	-0.8463
O	3.952364	10.39174	0.566098
O	3.659218	12.82483	3.751661
O	7.267723	5.482672	5.354666
O	6.040995	8.654205	7.527425
O	9.683854	10.39102	6.11615
O	9.977796	12.82098	2.928279
C	7.053856	6.39898	1.588109
C	7.734701	8.400316	0.294663
C	4.733602	11.10739	1.073411
C	4.521199	12.65567	2.96965
C	9.99826	8.670789	2.667228
C	10.25448	8.058899	1.409249
C	9.984742	6.666656	1.52248
C	9.556411	6.417547	2.862034
C	9.559178	7.655694	3.567372
C	7.438416	13.23868	0.570259
C	6.976609	14.22861	1.481271
C	5.618898	14.52081	1.16311
C	5.241332	13.7223	0.049651
C	6.368591	12.92304	-0.31135
C	6.584395	6.399575	5.092244
C	5.901568	8.400455	6.386449
C	8.902709	11.10672	5.608788
C	9.115768	12.65329	3.710774
C	3.638312	8.669873	4.013991
C	3.382288	8.057372	5.271718
C	3.652712	6.665289	5.157947
C	4.081216	6.416895	3.818313
C	4.077931	7.65534	3.113483
C	6.198372	13.23861	6.109698
C	6.66104	14.22832	5.198845
C	8.018705	14.52002	5.517601
C	8.395415	13.7214	6.631252

[{(CO)$_2$CpMn}$_2$BAu]$_2$ with dispersion

Au	6.932469	10.07047	1.920203
Au	6.704405	10.07075	4.760564
O	6.440081	5.502096	1.517156
O	7.74982	8.616136	-0.80423
O	3.893783	10.3208	0.651448
O	3.819703	12.55462	4.09245
O	7.19722	5.503902	5.164335
O	5.885208	8.617857	7.484893
O	9.74367	10.32122	6.029062
O	9.816844	12.55491	2.588559
C	7.122327	6.434314	1.717276
C	7.844632	8.375934	0.34266
C	4.655262	11.04543	1.170774
C	4.57673	12.46823	3.196416
C	9.798416	8.888432	2.897067
C	10.23666	8.26974	1.696779
C	10.06303	6.862901	1.824999
C	9.506856	6.613459	3.113924
C	9.336199	7.864859	3.774347
C	7.300568	13.09514	0.652513
C	6.953077	14.04226	1.653554
C	5.597059	14.42374	1.448585
C	5.103919	13.72097	0.316735
C	6.160501	12.89342	-0.1692
C	6.514527	6.435743	4.963984
C	5.791018	8.377461	6.337979
C	8.982014	11.04583	5.510003
C	9.059882	12.46866	3.484647
C	3.837795	8.888585	3.783054
C	3.399506	8.269906	4.983331
C	3.573732	6.863111	4.855375
C	4.130269	6.613681	3.566613
C	4.300602	7.865048	2.906045
C	6.335933	13.09383	6.029368
C	6.682538	14.04154	5.028563
C	8.038354	14.42385	5.233293
C	8.532259	13.72103	6.364767

C	7.267651	12.92257	6.991781
B	6.818351	8.152177	3.340486
B	6.818249	11.88867	3.340343
Mn	5.740913	12.44048	1.719253
Mn	8.16072	7.731144	1.918566
Mn	7.896068	12.43966	4.96145
Mn	5.476246	7.730787	4.762421
H	10.12886	9.718144	2.899426
H	10.59317	8.570888	0.516409
H	10.08769	5.928836	0.73647
H	9.281899	5.456385	3.276559
H	9.335396	7.791301	4.616089
H	8.432654	12.81387	0.547592
H	7.565108	14.69653	2.256143
H	4.986975	15.24343	1.665613
H	4.272542	13.71593	-0.4348
H	6.405148	12.19916	-1.11668
H	3.507574	9.717301	3.782178
H	3.043462	8.568852	6.164795
H	3.550113	5.927166	5.943719
H	4.356223	5.455999	3.403478
H	4.301694	7.791572	2.064846
H	5.203939	12.81425	6.13185
H	6.073042	14.69647	4.423747
H	8.651121	15.24242	5.015405
H	9.363939	13.71474	7.116229
H	7.230536	12.19875	7.797141

C	7.476358	12.8926	6.850699
B	6.818321	8.183076	3.340322
B	6.818487	11.79473	3.340453
Mn	5.664857	12.34976	1.836354
Mn	8.180451	7.798831	2.007192
Mn	7.971904	12.34994	4.844801
Mn	5.455927	7.799785	4.673478
H	9.788884	9.944518	3.097124
H	10.60822	8.788438	0.824737
H	10.28811	6.120714	1.073512
H	9.225354	5.653712	3.517882
H	8.967286	8.012591	4.77573
H	8.255264	12.60437	0.558897
H	7.604731	14.39631	2.432311
H	5.03502	15.12493	2.048886
H	4.103782	13.77799	-0.08715
H	6.097288	12.20116	-0.99624
H	3.847088	9.944642	3.582894
H	3.027546	8.788557	5.85523
H	3.348818	6.120948	5.606937
H	4.412282	5.653983	3.162887
H	4.669698	8.01276	1.904728
H	5.381568	12.60241	6.123068
H	6.030407	14.39553	4.250186
H	8.599713	15.1257	4.633136
H	9.532438	13.77862	6.768468
H	7.540282	12.20008	7.677473

$[\{(CO)_2CpMn\}_2BCu]_2$ without dispersion

Cu	10.35544	16.44941	3.281967
Cu	8.697267	18.27904	3.055855
O	8.002471	18.99922	5.972949
O	7.700556	14.85315	6.042224
O	7.281647	12.92061	3.249838
O	10.90908	15.89732	6.224251
O	11.8681	19.00633	-0.04353
O	7.737252	19.27889	0.363198
O	11.3576	13.50575	3.406043
O	11.25919	19.90623	6.268798
C	7.663531	18.19857	5.179968

$[\{(CO)_2CpMn\}_2BAu]_2$ dispersion

Cu	10.29852	16.42076	3.399325
Cu	8.673323	18.18454	2.910584
O	8.451097	18.80216	6.027508
O	7.841427	14.62563	5.794591
O	6.846282	13.31701	2.857463
O	10.67062	16.17629	6.334405
O	12.09412	19.16185	0.133762
O	7.818899	19.10257	0.201583
O	11.02561	13.35061	3.682856
O	11.44845	20.12103	6.05289
C	7.951595	18.06562	5.263792

C	7.420963	15.6916	5.277191
C	8.026462	13.67858	2.755702
C	11.29078	16.63963	5.398581
C	11.14215	19.40383	0.792906
C	8.643573	19.48267	1.095618
C	10.48794	14.09498	2.875323
C	11.55181	19.08912	5.480731
C	4.940371	17.85913	4.523682
C	5.411026	18.26321	3.236506
C	5.199725	15.96909	3.228823
C	5.564026	17.09409	2.436991
C	9.970148	15.74951	0.128543
C	4.81942	16.4478	4.521274
C	9.10116	13.61381	0.054281
C	8.012255	14.54174	0.075397
C	8.54982	15.8572	0.117077
C	9.687995	21.53931	3.599595
C	8.905405	21.88856	2.463472
C	9.784617	22.16385	1.374478
C	11.11514	21.97773	1.84283
C	11.05814	21.60026	3.215363
C	14.1419	18.52595	4.640445
C	13.77887	18.71924	3.27367
C	13.48992	17.44137	2.714521
C	13.67542	16.46433	3.733518
C	14.08749	17.13618	4.923848
C	10.3036	14.36461	0.087163
Mn	6.889833	16.95569	4.150027
Mn	9.138313	14.74956	1.901106
Mn	10.05529	20.10143	1.989532
Mn	12.11373	17.84758	4.355961
B	8.260385	16.07597	3.068155
B	10.79672	18.68023	3.168658
H	4.734113	18.51582	5.360128
H	5.588654	19.28149	2.914726
H	5.202555	14.93429	2.913783
H	5.866858	17.07871	1.399802
H	10.67032	16.57172	0.112529
H	4.494161	15.83526	5.35312
H	9.02033	12.53471	0.01595
H	6.96042	14.28489	0.038079

C	7.541428	15.57129	5.177282
C	7.794685	13.91934	2.522161
C	11.11962	16.81024	5.455329
C	11.23639	19.47007	0.875892
C	8.687958	19.33573	0.969368
C	10.27644	13.98551	3.038937
C	11.63141	19.21064	5.338459
C	5.374806	18.30797	4.212698
C	5.81013	18.08675	2.872931
C	5.185983	16.05893	3.774314
C	5.692595	16.68866	2.605666
C	9.991249	16.20217	0.504598
C	4.988916	17.06244	4.772064
C	9.749315	13.94615	0.076465
C	8.474953	14.56605	-0.04627
C	8.621591	15.95807	0.212676
C	9.619146	21.18928	3.709612
C	8.659711	21.50505	2.70932
C	9.349363	22.00764	1.56409
C	10.73594	21.99046	1.858121
C	10.90709	21.49286	3.182849
C	14.0745	18.30923	4.433264
C	13.61159	18.55027	3.104712
C	13.1365	17.31603	2.580082
C	13.30891	16.31687	3.580919
C	13.89825	16.93276	4.724421
C	10.68337	14.96144	0.428686
Mn	7.036227	16.95829	4.2165
Mn	9.172712	14.82807	1.934305
Mn	9.983208	20.01089	1.971183
Mn	12.01238	17.8471	4.306875
B	8.289227	16.06318	3.099165
B	10.7377	18.66662	3.143942
H	5.378986	19.25872	4.727
H	6.129087	18.83846	2.166969
H	5.021284	14.99902	3.890581
H	5.945152	16.19848	1.679235
H	10.41667	17.16199	0.735928
H	4.637867	16.89747	5.779818
H	9.962923	12.89476	-0.04623
H	7.548992	14.06397	-0.28826

H	7.988637	16.77783	0.087248
H	9.313525	21.29475	4.58395
H	7.823852	21.94477	2.431322
H	9.493222	22.45367	0.372314
H	12.01661	22.11636	1.25755
H	11.90478	21.42244	3.861716
H	14.42024	19.30453	5.340784
H	13.75306	19.66267	2.746107
H	13.21644	17.24931	1.686548
H	13.53463	15.3964	3.624776
H	14.30057	16.66921	5.877516
H	11.30702	13.95529	0.082898

H	7.846601	16.70383	0.179782
H	9.411623	20.78733	4.686753
H	7.591591	21.36888	2.794113
H	8.898629	22.30768	0.629307
H	11.52876	22.28757	1.186068
H	11.84352	21.3549	3.696919
H	14.47527	19.05234	5.107805
H	13.60096	19.49643	2.587427
H	12.73162	17.16995	1.593324
H	13.02813	15.27738	3.498083
H	14.12464	16.44343	5.660386
H	11.73528	14.81333	0.623772

{$(CO)_2$CpMn}$_2$B{Cu(PCy_3)}{Pt(PCy_3)}]
without dispersion

Mn	6.287589	6.960661	6.74922
Mn	6.70913	7.077202	2.918896
B	6.890715	6.700542	4.885858
O	7.563701	5.010592	8.592955
C	7.279923	5.711871	7.668762
O	4.00894	5.211083	6.18188
C	4.978744	5.856994	6.385287
O	8.682635	5.660192	1.267116
C	7.926956	6.144436	2.036841
O	8.618316	9.17798	3.660191
C	7.886359	8.292803	3.400032
C	6.675194	8.336058	8.420368
H	7.309969	8.088915	9.262211
C	7.09113	8.972069	7.217682
H	8.089916	9.309669	6.979264
C	5.959444	9.087	6.365626
H	5.951059	9.556413	5.393219
C	4.845301	8.516761	7.042202
H	3.827477	8.464088	6.675003
C	5.288052	8.051301	8.322008
H	4.675754	7.567442	9.072837
C	4.6103	6.512156	2.599164
H	4.118981	5.643583	3.011317
C	5.278232	6.602385	1.343489
H	5.415275	5.798181	0.631924
C	5.742418	7.938114	1.175363

{(CO)2CpMn}2B{Cu(PCy3)}{Pt(PCy3)}]
with dispersion

Mn	6.366275	7.108947	6.803667
Mn	6.631512	7.090449	3.050144
B	6.845865	6.774829	4.961521
O	7.576569	5.229126	8.741378
C	7.242279	5.854066	7.782785
O	3.96756	5.560998	6.187802
C	4.966142	6.134366	6.448388
O	8.242864	5.407217	1.26035
C	7.664645	5.998494	2.111356
O	8.634511	9.119402	3.695164
C	7.873001	8.260539	3.435069
C	7.096173	8.494541	8.265531
H	7.775724	8.2056	9.053452
C	7.471157	8.950857	6.973085
H	8.474559	9.074619	6.600224
C	6.282847	9.153398	6.219617
H	6.238869	9.492107	5.200017
C	5.176996	8.821758	7.050599
H	4.133797	8.8634	6.771686
C	5.678471	8.414366	8.324415
H	5.090823	8.079336	9.166283
C	4.588579	6.465397	2.920894
H	4.204551	5.557497	3.356109
C	5.124055	6.632827	1.611769
H	5.23866	5.858434	0.869425
C	5.536059	7.985368	1.460052

H	6.295472	8.321586	0.326826	H	6.009293	8.414655	0.589028
C	5.366701	8.66954	2.337301	C	5.25611	8.654331	2.685577
H	5.5708	9.718564	2.515918	H	5.470423	9.691691	2.898548
C	4.661646	7.792891	3.21036	C	4.666613	7.720484	3.581233
H	4.232646	8.049069	4.16615	H	4.361787	7.910849	4.593879
Pt	8.583301	5.997659	5.998525	Pt	8.46559	5.894509	6.02665
P	10.96603	5.755214	6.051831	P	10.67215	5.407695	5.811623
C	11.76907	7.09929	7.165109	C	11.59041	6.534574	6.973077
H	12.65771	6.601218	7.571334	H	12.48472	5.989553	7.290895
C	10.84984	7.474194	8.347164	C	10.71527	6.820296	8.196337
H	9.957175	7.966141	7.948626	H	9.830298	7.357047	7.844491
H	10.49249	6.58478	8.869641	H	10.34668	5.899286	8.641785
C	11.54913	8.409608	9.346571	C	11.45194	7.66535	9.226034
H	12.37968	7.875002	9.831043	H	12.3167	7.105682	9.605596
H	10.84698	8.674452	10.14789	H	10.79606	7.858317	10.0818
C	12.085	9.675683	8.670306	C	11.9301	8.975193	8.612647
H	11.24049	10.29317	8.331817	H	11.05673	9.572525	8.322488
H	12.64588	10.28618	9.389712	H	12.49325	9.562647	9.345455
C	12.96858	9.325656	7.4695	C	12.78153	8.710552	7.376958
H	13.89416	8.847938	7.823048	H	13.69978	8.189311	7.676433
H	13.27561	10.23903	6.9433	H	13.08526	9.654822	6.912452
C	12.26161	8.387795	6.474837	C	12.03455	7.856668	6.356762
H	11.41195	8.918806	6.029053	H	11.14705	8.398697	6.01914
H	12.95733	8.158434	5.663831	H	12.66563	7.686086	5.48333
C	11.92842	5.848837	4.410335	C	11.42993	5.630652	4.150609
H	12.96705	6.05271	4.704581	H	12.50925	5.732126	4.305127
C	11.4456	6.994332	3.497072	C	10.89415	6.871991	3.446334
H	11.4119	7.946625	4.023647	H	10.95074	7.754024	4.073838
H	10.42052	6.783454	3.189366	H	9.836627	6.70293	3.253399
C	12.33257	7.13687	2.249141	C	11.6402	7.105042	2.138605
H	11.93648	7.943461	1.619133	H	11.24076	7.996781	1.644481
H	13.34352	7.449379	2.551327	H	12.69584	7.308184	2.362779
C	12.41584	5.833793	1.446612	C	11.53347	5.900209	1.211259
H	13.13838	5.937378	0.626415	H	12.1699	6.040071	0.330425
H	11.44277	5.63431	0.983129	H	10.50466	5.81932	0.859448
C	12.80485	4.652043	2.341341	C	11.91155	4.605452	1.922586
H	13.85302	4.760583	2.656961	H	12.98856	4.605262	2.136671
H	12.75234	3.714448	1.772967	H	11.71724	3.750908	1.269274
C	11.91634	4.536706	3.594919	C	11.16343	4.433485	3.241972
H	10.88631	4.312098	3.296329	H	10.08744	4.382122	3.068325
H	12.26391	3.687532	4.187523	H	11.45806	3.49651	3.71051

C	11.63868	4.142326	6.826475	C	11.27035	3.734913	6.327613
H	11.24894	3.388992	6.128231	H	10.68351	3.049092	5.711195
C	13.17537	3.992884	6.885998	C	12.75327	3.476413	6.073103
H	13.59317	4.732512	7.579899	H	13.34643	4.147684	6.701773
H	13.63838	4.186803	5.915074	H	13.0203	3.691257	5.038993
C	13.59257	2.592311	7.370831	C	13.10974	2.03196	6.412966
H	13.29401	1.844001	6.62217	H	12.55873	1.363327	5.739512
H	14.68762	2.542477	7.435274	H	14.17626	1.858753	6.233042
C	12.96305	2.240636	8.723057	C	12.75072	1.699391	7.855791
H	13.22085	1.211316	9.004541	H	12.97533	0.649549	8.072045
H	13.38948	2.890118	9.501511	H	13.37336	2.305513	8.526151
C	11.44104	2.419049	8.689319	C	11.28271	1.996901	8.134288
H	11.01507	2.239575	9.684874	H	11.04709	1.801234	9.18535
H	11.00284	1.662127	8.024905	H	10.66024	1.324662	7.536206
C	11.03134	3.820596	8.206935	C	10.93518	3.439637	7.78668
H	9.943192	3.906905	8.176228	H	9.885043	3.653386	7.985051
H	11.38275	4.551435	8.944772	H	11.52541	4.103999	8.423272
Cu	7.073072	4.731977	3.968425	Cu	7.246295	4.886642	3.964276
P	6.678317	2.395	3.847934	P	7.08158	2.695818	3.982913
C	8.015852	1.448373	2.879456	C	8.563593	1.728685	3.446326
H	8.839348	1.464665	3.605293	H	9.379761	2.143243	4.043354
C	8.550905	2.170569	1.626808	C	8.894566	1.971683	1.975547
H	8.804209	3.205433	1.860148	H	8.955983	3.039104	1.770367
H	7.781621	2.212592	0.848515	H	8.083138	1.573024	1.360847
C	9.782502	1.449134	1.054269	C	10.18805	1.263813	1.588495
H	10.61291	1.522019	1.770538	H	11.0155	1.690738	2.162441
H	10.11308	1.966983	0.145108	H	10.40503	1.442934	0.530497
C	9.502939	-0.0277	0.752933	C	10.10332	-0.23067	1.8721
H	8.783495	-0.10143	-0.0756	H	9.325997	-0.67484	1.237587
H	10.41884	-0.52632	0.411116	H	11.04728	-0.72289	1.615408
C	8.936882	-0.75005	1.980816	C	9.754355	-0.48236	3.333862
H	9.713942	-0.81772	2.75577	H	10.57125	-0.11406	3.967473
H	8.666181	-1.78309	1.724925	H	9.657876	-1.55604	3.527381
C	7.705415	-0.02799	2.554993	C	8.462735	0.230363	3.725455
H	6.898205	-0.0792	1.814494	H	7.637396	-0.18874	3.141769
H	7.349508	-0.56468	3.440196	H	8.237419	0.04299	4.774629
C	5.033533	1.883642	3.018648	C	5.766084	2.054124	2.84198
H	5.114778	0.795191	2.901659	H	6.128195	1.089098	2.475738
C	4.879051	2.509292	1.617263	C	5.633642	3.014102	1.66086
H	5.722998	2.239478	0.979358	H	6.604899	3.222669	1.215805
H	4.899017	3.599556	1.709684	H	5.27257	3.964568	2.054809

C	3.576873	2.081403	0.918343
H	3.500267	2.599281	-0.04666
H	3.624466	1.006861	0.687991
C	2.337834	2.359994	1.77436
H	1.437504	1.981618	1.273665
H	2.199306	3.445171	1.88086
C	2.482215	1.724717	3.158973
H	1.62467	1.986333	3.791995
H	2.468974	0.62946	3.058531
C	3.775652	2.156241	3.87251
H	3.711353	3.220654	4.123586
H	3.835179	1.616868	4.819875
C	6.59932	1.435924	5.501148
H	5.978855	0.556674	5.275809
C	5.907978	2.261754	6.605843
H	4.950148	2.659428	6.275029
H	6.532901	3.128364	6.830664
C	5.70191	1.446198	7.89167
H	5.231541	2.086995	8.647463
H	4.997211	0.623203	7.699596
C	7.021567	0.880679	8.424353
H	7.646422	1.708163	8.783776
H	6.839697	0.228185	9.288225
C	7.770053	0.10938	7.33309
H	7.218996	-0.81196	7.093025
H	8.754488	-0.21082	7.698041
C	7.953639	0.928608	6.041109
H	8.606392	1.787168	6.241714
H	8.468306	0.299107	5.311831

{(CO)$_2$CpMn}$_2$B{Cu(ITol)}{Pt(PCy$_3$)}]
without dispersion

Pt	7.308311	1.493381	4.291855
Mn	9.759415	1.309359	5.881208
P	6.844125	3.256043	2.762623
B	8.051362	0.33368	5.872384
O	10.93763	-1.31679	5.323704
C	10.42937	-0.27323	5.549484
Cu	7.321874	-0.5418	7.790088
Mn	6.40038	-0.69807	5.502085

C	4.661084	2.477896	0.618183
H	4.563649	3.193751	-0.20528
H	5.068039	1.553044	0.189456
C	3.297217	2.187935	1.234184
H	2.623021	1.75602	0.487236
H	2.847352	3.131967	1.565972
C	3.428088	1.256018	2.433404
H	2.449345	1.08817	2.895396
H	3.79697	0.279124	2.095847
C	4.395229	1.823802	3.468284
H	4.001712	2.777056	3.831224
H	4.466485	1.15425	4.326713
C	6.712636	2.035021	5.654731
H	6.368786	1.002646	5.534442
C	5.632565	2.867465	6.337701
H	4.756487	2.997376	5.711174
H	6.049676	3.86063	6.501951
C	5.240782	2.237957	7.667853
H	4.468206	2.8481	8.146014
H	4.800992	1.249752	7.477356
C	6.44539	2.099556	8.590759
H	6.759439	3.094604	8.904849
H	6.170421	1.5433	9.493738
C	7.616759	1.413892	7.895642
H	7.375103	0.356858	7.720419
H	8.496537	1.435908	8.542841
C	7.94565	2.058835	6.552761
H	8.236541	3.104795	6.680983
H	8.788522	1.543918	6.094642

{(CO)$_2$CpMn}$_2$B{Cu(ITol)}{Pt(PCy$_3$)}]
with dispersion

Pt	7.485754	1.508704	4.489575
Mn	9.078792	1.865788	6.631013
P	6.710268	3.087914	3.018777
B	8.12828	0.328734	6.084
O	11.37727	0.140499	6.189053
C	10.43549	0.836874	6.34099
Cu	7.279841	-0.88252	7.486118
Mn	7.226307	-1.05984	5.068857

O	4.425984	0.64102	7.231789
C	5.271975	0.09706	6.611148
O	10.50001	1.971519	3.114644
C	9.99461	1.703125	4.149592
O	4.48735	0.685909	3.698146
C	5.549819	0.555278	4.244665
C	9.305363	3.109904	7.063585
H	8.403212	3.698168	6.971198
C	10.52216	3.295735	6.342184
H	10.70788	4.055506	5.595579
C	11.4578	2.317989	6.775939
H	12.4702	2.195637	6.411052
C	10.81029	1.510679	7.756414
H	11.25523	0.67885	8.28129
C	9.488954	2.009474	7.942731
H	8.758126	1.637237	8.647668
C	6.506463	-2.75544	6.109488
H	6.576248	-3.12539	7.124395
C	7.606807	-2.49246	5.232612
H	8.656991	-2.58169	5.470802
C	7.075367	-2.11893	3.972126
H	7.652751	-1.83416	3.102214
C	5.650535	-2.16107	4.06161
H	4.955847	-1.91567	3.26983
C	5.297851	-2.5589	5.372456
H	4.290171	-2.67843	5.75109
C	5.037344	3.78693	2.975689
H	4.554601	2.825231	2.76887
C	4.656216	4.124234	4.434976
H	5.059108	5.103453	4.717981
H	5.094351	3.390516	5.116739
C	3.128751	4.149625	4.604973
H	2.735926	3.137107	4.445557
H	2.879271	4.420375	5.639854
C	2.459491	5.124762	3.628263
H	1.366757	5.0618	3.717534
H	2.732048	6.156286	3.898228
C	2.884535	4.855583	2.178703
H	2.461438	5.619262	1.512041
H	2.468572	3.892388	1.850594
C	4.416647	4.822826	2.017604

O	4.627276	-1.67192	6.254357
C	5.707793	-1.36496	5.882531
O	9.962827	3.346053	4.265641
C	9.331817	2.660167	5.004132
O	5.15369	-0.00408	3.282635
C	6.139965	0.055021	3.955514
C	7.739404	3.231547	7.616642
H	6.801197	3.546748	7.190891
C	9.010416	3.854757	7.429825
H	9.201066	4.737495	6.840701
C	9.984808	3.085232	8.111036
H	11.04723	3.278631	8.13287
C	9.317069	1.9684	8.710257
H	9.784456	1.186987	9.290527
C	7.927954	2.075954	8.411102
H	7.149952	1.410691	8.728005
C	8.148497	-2.92318	5.314614
H	8.131992	-3.49474	6.229781
C	9.136991	-1.9597	4.951409
H	9.989987	-1.66984	5.535063
C	8.794041	-1.44388	3.673637
H	9.316557	-0.65675	3.153287
C	7.593446	-2.08353	3.243252
H	7.056579	-1.8783	2.33013
C	7.200004	-3.00288	4.247651
H	6.312014	-3.61752	4.230703
C	4.878252	3.086241	3.092414
H	4.661948	2.092595	2.692829
C	4.36109	3.065943	4.531172
H	4.518962	4.042621	4.99519
H	4.934426	2.336591	5.106509
C	2.876277	2.730722	4.542059
H	2.746782	1.714438	4.154011
H	2.494562	2.737162	5.567962
C	2.083655	3.71108	3.684239
H	1.023968	3.434601	3.668332
H	2.146764	4.710438	4.1347
C	2.630949	3.776486	2.262461
H	2.078351	4.519555	1.676965
H	2.482127	2.805272	1.775405
C	4.120952	4.106785	2.257058

H	4.661409	4.602776	0.973522
H	4.812176	5.822809	2.230852
C	6.941911	2.692791	0.94908
H	6.614138	3.550695	0.348023
C	6.00719	1.50776	0.626435
H	6.284821	0.654413	1.254344
H	4.968186	1.743104	0.871199
C	6.092596	1.100709	-0.85423
H	5.694598	1.912708	-1.481
H	5.441928	0.232924	-1.02625
C	7.527702	0.78273	-1.28817
H	7.869795	-0.12658	-0.77315
H	7.562177	0.562747	-2.36342
C	8.472923	1.939077	-0.94862
H	8.228357	2.807615	-1.57845
H	9.509776	1.667191	-1.18739
C	8.384727	2.335423	0.53281
H	9.070929	3.16458	0.720045
H	8.739721	1.505108	1.151292
C	7.985785	4.784614	2.789243
H	8.969727	4.299198	2.751923
C	7.91558	5.779489	1.609486
H	6.948747	6.293802	1.607541
H	7.996262	5.264056	0.649469
C	9.034657	6.83328	1.69886
H	10.00864	6.339027	1.572136
H	8.936319	7.53953	0.863533
C	9.017683	7.585992	3.033916
H	9.868101	8.277944	3.091116
H	8.110336	8.205076	3.091916
C	9.044813	6.611981	4.217482
H	8.952062	7.161684	5.163764
H	10.0209	6.108612	4.24543
C	7.925165	5.56215	4.120479
H	7.980911	4.869734	4.963363
H	6.963632	6.080581	4.201837
C	7.44114	-1.04948	9.678276
N	6.379823	-1.15109	10.55471
C	6.782514	-1.53851	11.82498
H	6.081404	-1.67977	12.63393
C	8.125169	-1.69834	11.77556

H	4.500327	4.130114	1.23288
H	4.265425	5.104077	2.679903
C	7.123511	2.735106	1.259412
H	6.642256	3.497929	0.639942
C	6.62233	1.362666	0.827932
H	7.062815	0.620364	1.495164
H	5.542158	1.287973	0.950898
C	7.014904	1.054485	-0.61051
H	6.527793	1.771482	-1.28368
H	6.649456	0.05923	-0.88626
C	8.523393	1.138955	-0.79746
H	9.002736	0.363471	-0.1869
H	8.79408	0.941117	-1.84
C	9.041208	2.502726	-0.36187
H	8.631333	3.271427	-1.0295
H	10.13165	2.546979	-0.45535
C	8.637812	2.819281	1.072016
H	9.009369	3.80441	1.352713
H	9.103173	2.106327	1.758451
C	7.359574	4.792582	3.243279
H	8.434725	4.606683	3.263581
C	7.095931	5.822679	2.151392
H	6.034326	6.071509	2.115854
H	7.366812	5.427085	1.171761
C	7.901629	7.088411	2.433971
H	8.97002	6.847102	2.377589
H	7.700684	7.839642	1.662297
C	7.591826	7.653995	3.8155
H	8.206892	8.539283	4.008698
H	6.543791	7.979573	3.841216
C	7.812083	6.606588	4.900914
H	7.548766	7.013193	5.883571
H	8.874617	6.340403	4.932695
C	7.00167	5.350201	4.614399
H	7.184332	4.583846	5.364159
H	5.936872	5.595432	4.642759
C	7.215144	-1.223	9.352335
N	6.398575	-0.69103	10.31478
C	6.694081	-1.19048	11.57466
H	6.15059	-0.89034	12.45505
C	7.717582	-2.07204	11.41039

H	8.826095	-2.01966	12.53095
N	8.516226	-1.40592	10.47315
C	4.976465	-0.95756	10.26371
C	4.236632	-2.00159	9.709909
H	4.728214	-2.933	9.442366
C	2.866673	-1.84443	9.510408
H	2.30054	-2.66413	9.071713
C	2.209756	-0.65786	9.863685
C	2.973372	0.368388	10.43683
H	2.492992	1.302277	10.7219
C	4.345002	0.227293	10.63879
H	4.923516	1.036747	11.07712
C	0.736195	-0.47562	9.59432
H	0.270654	0.187067	10.33036
H	0.202597	-1.43102	9.606188
H	0.583201	-0.02607	8.604296
C	9.899915	-1.5294	10.0903
C	10.28308	-2.35805	9.034553
H	9.530149	-2.87446	8.447916
C	11.63484	-2.52185	8.741994
H	11.91502	-3.15635	7.904189
C	12.63245	-1.88677	9.496861
C	12.22416	-1.06464	10.55508
H	12.96916	-0.54459	11.15439
C	10.87433	-0.87878	10.85088
H	10.58189	-0.21728	11.66266
C	14.09312	-2.08338	9.171162
H	14.40424	-3.11714	9.367808
H	14.72984	-1.42409	9.76802
H	14.29191	-1.88252	8.112331

{(CO)$_2$CpMn}$_2$B{Cu(IMe)}{Pt(PCy$_3$)}]
without dispersion

Mn	6.159797	5.970676	6.99574
Mn	6.479844	8.27701	3.933296
B	6.676617	6.879546	5.348187
O	7.503287	3.331486	7.245825
C	7.217832	4.454655	6.953754
O	4.049808	4.72665	5.39964
C	4.939845	5.21273	6.006055

H	8.258239	-2.67389	12.12169
N	8.021979	-2.0714	10.05561
C	5.47309	0.34955	10.01802
C	4.7336	0.271044	8.841409
H	4.745249	-0.63341	8.25246
C	4.004805	1.373257	8.418546
H	3.462656	1.313273	7.480974
C	3.977358	2.553477	9.168791
C	4.672856	2.582429	10.38074
H	4.662617	3.487077	10.98115
C	5.42996	1.494305	10.80617
H	6.03478	1.561503	11.7037
C	3.250756	3.762113	8.658393
H	3.159159	4.528175	9.431012
H	2.250018	3.500073	8.303975
H	3.78927	4.196552	7.80912
C	9.083616	-2.79838	9.445588
C	9.273123	-4.14462	9.746444
H	8.609483	-4.64271	10.44536
C	10.294	-4.84643	9.114581
H	10.43307	-5.90013	9.34009
C	11.13107	-4.22404	8.181324
C	10.923	-2.87116	7.904522
H	11.56392	-2.35884	7.196799
C	9.90898	-2.15335	8.528969
H	9.757121	-1.10612	8.300864
C	12.22027	-4.99607	7.496837
H	12.94701	-5.37492	8.223505
H	12.7505	-4.37236	6.774844
H	11.80915	-5.8619	6.967281

{(CO)$_2$CpMn}$_2$B{Cu(IMe)}{Pt(PCy$_3$)}]
without dispersion

Mn	6.031203	5.869388	6.79264
Mn	6.926799	8.275812	4.025692
B	6.872041	6.855541	5.36074
O	7.283299	3.241026	7.347481
C	6.995769	4.338905	6.975803
O	4.276722	4.863038	4.682668
C	5.012149	5.238089	5.529358

O	8.867432	8.122442	2.224085	O	9.299553	7.980039	2.315969
C	7.919655	8.072953	2.931212	C	8.382452	8.015539	3.064331
O	8.081735	9.740849	5.895353	O	8.197353	9.563702	6.320414
C	7.457629	9.110336	5.124103	C	7.740197	9.015075	5.384715
C	6.54784	6.107317	9.151655	C	6.151588	6.049058	8.917999
H	7.236812	5.45845	9.678412	H	6.742958	5.381227	9.527733
C	6.869387	7.360969	8.558034	C	6.611273	7.257294	8.326605
H	7.838122	7.842277	8.559888	H	7.602256	7.675349	8.402815
C	5.690904	7.872898	7.94484	C	5.547206	7.795362	7.550145
H	5.604611	8.821397	7.43586	H	5.588642	8.704521	6.976819
C	4.64472	6.930101	8.156951	C	4.434696	6.916745	7.665346
H	3.617249	7.030859	7.828232	H	3.474026	7.039537	7.185834
C	5.174351	5.833268	8.91081	C	4.804684	5.83192	8.517983
H	4.62975	4.952981	9.229402	H	4.185985	4.987891	8.785148
C	4.593645	7.783927	2.927926	C	5.14893	7.729442	2.952328
H	4.303946	6.80636	2.566302	H	4.904983	6.728841	2.633078
C	5.27797	8.794159	2.187515	C	5.874171	8.712865	2.218177
H	5.63194	8.70629	1.167425	H	6.304849	8.578979	1.237001
C	5.431007	9.937159	3.019187	C	5.974325	9.888857	3.010831
H	5.917442	10.86641	2.749048	H	6.485933	10.80054	2.73926
C	4.838251	9.629225	4.282162	C	5.315483	9.624858	4.245942
H	4.785865	10.29703	5.133612	H	5.2333	10.3132	5.074791
C	4.317995	8.305861	4.224107	C	4.803738	8.298258	4.20803
H	3.801285	7.785407	5.016601	H	4.279518	7.790124	4.998052
Pt	8.447159	5.698677	5.809988	Pt	8.424318	5.555958	5.948004
P	10.74388	5.197049	5.595271	P	10.64419	5.240117	5.549882
C	11.95926	6.648804	5.322642	C	11.67617	6.7583	5.283414
H	12.78736	6.413638	6.002516	H	12.52256	6.654773	5.968501
C	11.33721	7.983786	5.780571	C	10.85508	7.974633	5.699109
H	10.51395	8.240732	5.108059	H	9.999822	8.038278	5.030363
H	10.89447	7.891977	6.774974	H	10.44228	7.835415	6.69771
C	12.36724	9.123489	5.779206	C	11.67114	9.256482	5.619904
H	13.13354	8.928053	6.544279	H	12.48834	9.213704	6.352327
H	11.87032	10.05739	6.070789	H	11.0382	10.10634	5.889894
C	13.04119	9.285823	4.412996	C	12.25728	9.445366	4.225845
H	12.30142	9.648898	3.685804	H	11.4381	9.57625	3.510219
H	13.82868	10.04915	4.463484	H	12.87293	10.35039	4.187833
C	13.62359	7.957385	3.919821	C	13.07956	8.228488	3.815905
H	14.47245	7.669523	4.557707	H	13.9438	8.130215	4.485789
H	14.02668	8.072315	2.904774	H	13.47162	8.357372	2.800977
C	12.5798	6.826774	3.923527	C	12.23832	6.956441	3.879956

H	11.79613	7.071583	3.197571
H	13.06089	5.904334	3.584709
C	11.24015	3.827972	4.375743
H	12.33849	3.817749	4.355038
C	10.72575	4.107048	2.948667
H	11.0623	5.079608	2.587724
H	9.632729	4.15486	2.978095
C	11.16809	3.02149	1.955135
H	10.75678	3.248529	0.962497
H	12.26236	3.047678	1.84563
C	10.73532	1.621236	2.399621
H	11.13584	0.861389	1.715602
H	9.640692	1.545771	2.341412
C	11.18761	1.333789	3.834217
H	12.28322	1.238665	3.856832
H	10.78976	0.367774	4.172506
C	10.75417	2.430977	4.820379
H	9.661291	2.440669	4.905319
H	11.13447	2.173486	5.811134
C	11.32751	4.499085	7.268569
H	10.65964	3.636735	7.388486
C	12.78274	3.993768	7.35263
H	13.4793	4.835016	7.245961
H	13.01375	3.298083	6.541024
C	13.05818	3.300982	8.69934
H	12.46137	2.379667	8.760054
H	14.11063	2.990076	8.740065
C	12.72445	4.201023	9.893809
H	12.85947	3.648272	10.83256
H	13.43416	5.040884	9.925009
C	11.29608	4.748307	9.799169
H	11.10705	5.455419	10.61759
H	10.57873	3.925517	9.92821
C	11.03305	5.441745	8.453491
H	9.996391	5.786037	8.405871
H	11.6677	6.33418	8.389636
Cu	7.063243	5.875888	3.437445
C	6.831644	4.42796	2.145261
N	6.388015	3.144765	2.365682
C	6.202401	2.45144	1.177043
C	6.545957	3.297427	0.172234

H	11.41259	7.062678	3.179029
H	12.83054	6.090528	3.574636
C	11.04741	3.987154	4.259259
H	12.13231	3.947023	4.120761
C	10.36326	4.367349	2.949139
H	10.72424	5.323633	2.581522
H	9.300305	4.501506	3.167259
C	10.52168	3.297814	1.878252
H	9.988088	3.609289	0.974264
H	11.58114	3.198695	1.608945
C	9.99585	1.955077	2.365028
H	10.14236	1.183339	1.601739
H	8.920725	2.042549	2.533614
C	10.68146	1.552788	3.664079
H	11.748	1.383457	3.468853
H	10.27232	0.60721	4.037652
C	10.53524	2.626948	4.73646
H	9.482883	2.744164	5.009458
H	11.06256	2.31484	5.638263
C	11.34931	4.545692	7.108262
H	10.74239	3.655193	7.297562
C	12.81608	4.139272	7.033031
H	13.4258	5.017992	6.800764
H	12.97271	3.420601	6.225833
C	13.28418	3.548625	8.361006
H	12.73952	2.614238	8.546289
H	14.34815	3.294703	8.299945
C	13.03047	4.508687	9.51851
H	13.34736	4.055077	10.46334
H	13.63845	5.411596	9.375841
C	11.56022	4.90673	9.584827
H	11.39348	5.618492	10.40054
H	10.95312	4.018958	9.800854
C	11.10008	5.512604	8.265359
H	10.03665	5.767474	8.294321
H	11.64975	6.441399	8.086779
Cu	7.375975	5.886192	3.567193
C	6.959173	4.441965	2.417343
N	6.585005	3.175645	2.769968
C	6.203124	2.42528	1.667005
C	6.349616	3.227308	0.579471

N	6.930955	4.490607	0.771384	N	6.813883	4.449999	1.054979
C	7.366944	5.660281	0.008556	C	7.116272	5.610234	0.22924
C	6.127446	2.557939	3.682968	C	6.62037	2.687355	4.142048
H	7.872308	6.354577	0.674284	H	7.598277	6.348643	0.865567
H	6.507087	6.156216	-0.45423	H	6.196392	6.026212	-0.19073
H	8.062711	5.344931	-0.77395	H	7.792545	5.322741	-0.57875
H	6.557223	3.150979	-0.89797	H	6.171881	3.041326	-0.46757
H	5.856644	1.428611	1.147812	H	5.87464	1.402472	1.753672
H	6.508212	1.533619	3.701289	H	7.14409	1.731759	4.175263
H	5.056259	2.559201	3.896762	H	5.610487	2.586409	4.535896
H	6.64324	3.145619	4.437165	H	7.163439	3.42585	4.725542

Table S7. Optimized structures used for thermochemical calculations. For the methods employed see the **Computational Details**. Columns are multi-page.

$[\{(CO)_2CpMn\}_2B\{Au(DMS)\}]$				$[\{(CO)_2CpMn\}_2BAu]$			
Au	1.310159	0.404629	0.015781	Au	0.697373	-1.64932	0.232982
Mn	-1.39012	-1.8947	0.175498	Mn	-2.19912	0.4204	0.123489
Mn	-0.85343	1.861174	-0.19675	Mn	1.638314	0.698406	-0.25715
B	-0.85278	-0.12673	-0.0041	B	-0.37321	0.256609	-0.03355
O	0.187027	-2.12518	2.636472	O	-1.84157	0.196267	3.025933
C	-0.44671	-2.02368	1.667473	C	-1.98528	0.287482	1.880287
O	-3.73551	-0.81656	1.553252	O	-1.78712	3.318419	0.155053
C	-2.80114	-1.25173	1.02108	C	-1.95934	2.172563	0.151649
O	-2.65761	0.975282	-2.32181	O	0.334679	1.747112	-2.66297
C	-1.92438	1.290046	-1.48856	C	0.80496	1.302281	-1.71312
O	0.945982	2.943533	-2.27709	O	3.380793	-0.89185	-2.06241
C	0.290314	2.466237	-1.45812	C	2.677847	-0.32406	-1.35808
C	-0.96511	-2.23425	-1.93643	C	-2.53224	-0.84163	-1.62616
H	-0.54772	-1.49612	-2.60328	H	-1.75424	-1.18207	-2.29141
C	-0.23149	-3.17992	-1.16736	C	-3.01122	-1.52882	-0.47459
H	0.841774	-3.28671	-1.14767	H	-2.66115	-2.48109	-0.10773
C	-1.16891	-3.99952	-0.46665	C	-4.06797	-0.75136	0.087373
H	-0.9261	-4.80467	0.209624	H	-4.63033	-0.99899	0.974677
C	-2.46942	-3.55609	-0.79772	C	-4.24386	0.403188	-0.71071
H	-3.39308	-3.96138	-0.41424	H	-4.95827	1.192931	-0.53776
C	-2.3499	-2.45824	-1.70383	C	-3.28291	0.356801	-1.76705
H	-3.16183	-1.90132	-2.14399	H	-3.16054	1.092707	-2.54655
C	-0.35394	2.758278	1.758848	C	2.423855	0.869905	1.809074
C	-1.47947	1.892422	1.888624	C	1.324256	1.755479	1.618725
C	-2.51924	2.39785	1.06276	C	1.674632	2.671218	0.584724
C	-2.03457	3.569473	0.407308	C	2.976915	2.335434	0.118085

C	-0.70148	3.790258	0.848953
H	0.590585	2.651829	2.267571
H	-1.5439	1.026627	2.527491
H	-3.49924	1.960213	0.953927
H	-2.58694	4.187954	-0.28261
H	-0.05567	4.59436	0.530008
S	3.640085	-0.40889	0.391438
C	4.28934	-0.86756	-1.25571
H	5.267249	-1.33489	-1.13613
H	3.602199	-1.53929	-1.76807
H	4.393312	0.055196	-1.82484
C	3.448155	-2.06083	1.153671
H	4.436203	-2.48648	1.330828
H	2.925568	-1.91984	2.098431
H	2.854729	-2.71019	0.512314

[{(CO)$_2$CpMn}$_2$B{Cu(DMS)}]

Mn	-2.03727	0.414627	0.193204
Mn	1.673493	1.153044	-0.15148
B	-0.19549	0.850112	0.029993
O	-2.12076	-1.98538	1.90811
C	-1.98482	-1.06517	1.206433
O	-1.80232	2.112914	2.574148
C	-1.89227	1.432536	1.640392
O	0.752321	2.974405	-2.25896
C	1.115625	2.244446	-1.43579
O	2.542813	-0.78609	-2.20095
C	2.118736	-0.08445	-1.37316
C	-2.33527	1.228914	-1.80944
H	-1.58273	1.729482	-2.39801
C	-2.64293	-0.16062	-1.84072
H	-2.15734	-0.90504	-2.45262
C	-3.73177	-0.38657	-0.94946
H	-4.19414	-1.34112	-0.74736
C	-4.10149	0.85368	-0.37212
H	-4.89167	1.011998	0.345369
C	-3.23288	1.853842	-0.90096
H	-3.25731	2.906275	-0.66251
C	2.565741	0.545951	1.76311
C	1.727458	1.678229	1.966312
C	2.250424	2.751318	1.194804
C	3.416789	2.286551	0.516691
C	3.606347	0.927251	0.866082

C	3.43639	1.222807	0.885434
H	2.465374	0.05866	2.518211
H	0.416087	1.767694	2.19858
H	1.049876	3.471894	0.219918
H	3.52766	2.84689	-0.65564
H	4.388756	0.727106	0.772739

[{(CO)$_2$CpMn}$_2$BCu]

Mn	-1.89094	0.187028	0.194542
Mn	1.891049	0.187011	-0.1946
B	0.000052	0.42345	-7.8E-05
O	-2.37136	-2.51904	1.314895
C	-2.05004	-1.49698	0.84827
O	-1.44546	1.304423	2.876125
C	-1.61917	0.845358	1.828788
O	1.445467	1.305646	-2.87565
C	1.619233	0.846069	-1.82854
O	2.371213	-2.51862	-1.31606
C	2.050091	-1.49672	-0.84898
C	-2.08899	1.231911	-1.70515
H	-1.27238	1.572703	-2.32262
C	-2.74982	-0.02138	-1.81317
H	-2.50769	-0.80793	-2.51112
C	-3.80374	-0.04795	-0.85131
H	-4.48645	-0.86735	-0.68463
C	-3.79593	1.183301	-0.15287
H	-4.46924	1.467802	0.641034
C	-2.73155	1.977157	-0.67471
H	-2.46923	2.97453	-0.35687
C	2.749747	-0.02209	1.813123
C	2.088757	1.231158	1.705592
C	2.731269	1.976944	0.675539
C	3.795827	1.183476	0.153456
C	3.803783	-0.04806	0.851377

H	2.451765	-0.41881	2.232698
H	0.869437	1.729278	2.617964
H	1.839633	3.747978	1.141751
H	4.038359	2.865267	-0.14891
H	4.4012	0.288588	0.511003
Cu	0.178625	-1.10397	-0.11544
S	0.517704	-3.36462	-0.43324
C	0.143258	-4.2519	1.122068
H	0.297003	-5.31949	0.963494
H	0.77717	-3.89218	1.932213
H	-0.89836	-4.05448	1.366002
C	2.307572	-3.71143	-0.5813
H	2.841104	-3.36451	0.303506
H	2.450271	-4.78391	-0.7166
H	2.66456	-3.1747	-1.45803

H	2.507767	-0.80891	2.51081
H	1.271949	1.571495	2.323039
H	2.468864	2.974436	0.358149
H	4.469217	1.468448	-0.64021
H	4.486606	-0.8673	0.684389
Cu	0.000066	-1.54409	-0.00011

[{(CO)$_2$CpMn}$_2$BCu]$_2$

Cu	-0.02108	-1.25342	-0.16194
O	0.02318	2.507538	-2.6709
C	0.843999	2.052551	-1.98758
Mn	2.313749	1.483257	-1.10499
B	1.880055	-0.11265	-0.0755
Cu	0.08203	1.255015	0.022697
O	2.803881	-0.51014	-3.20875
C	2.580211	0.250525	-2.37372
Mn	2.146958	-1.71739	1.015725
B	-1.82891	0.09286	0.004424
Mn	-2.12593	1.652499	1.168141
O	4.610758	-1.80128	-0.57272
C	3.617836	-1.76559	0.015231
C	-0.98922	-1.73572	-2.26851
O	-0.25195	-1.99014	-3.12055
C	-2.45418	0.338653	2.313983
O	-2.68193	-0.50296	3.077564
C	-0.61587	2.039829	2.058251
O	0.241661	2.392771	2.762693
Mn	-2.33615	-1.43119	-1.10097
O	1.118192	-4.06209	-0.45934
C	1.456263	-3.08251	0.060111
C	-2.87966	-0.1048	-2.15255
O	-3.28168	0.720381	-2.85458
C	3.030887	3.560268	-1.0259
H	2.50131	4.393384	-1.46345

[{(CO)$_2$CpMn}$_2$BAu]$_2$

Au	0.005918	-1.44773	-0.01653
O	4.655676	-1.75058	0.605636
C	3.741195	-1.64214	-0.08997
B	1.958012	0.000036	0.000006
O	1.601178	-4.219	-0.34094
Mn	-2.37033	-1.48084	1.229949
B	-1.81966	0	0.000006
C	1.810075	-3.11587	-0.61831
Mn	2.383232	-1.50545	-1.21643
C	-1.02385	-1.97941	2.314519
O	-0.28188	-2.3451	3.120283
O	-2.70711	0.875829	2.923718
C	-2.56018	-0.04083	2.233061
C	1.378646	-1.01797	-3.11914
H	0.317734	-0.88743	-3.25536
C	2.093156	-2.23667	-3.26471
H	1.6604	-3.19582	-3.50582
C	3.470586	-1.98135	-3.0266
H	4.267974	-2.70716	-3.06315
C	3.609377	-0.59353	-2.73032
H	4.52964	-0.07701	-2.50839
C	2.317279	0.000493	-2.78294
H	2.091447	1.046207	-2.65135
C	-3.1938	-2.86557	-0.28102
H	-2.75295	-3.03913	-1.24893
C	-4.16028	-1.86853	0.02411

C	2.868022	3.075685	0.302494
H	2.225107	3.491756	1.062406
C	4.433112	1.759149	-0.75861
H	5.161999	0.987621	-0.95356
C	3.742552	1.963018	0.465083
H	3.878721	1.394003	1.369726
C	1.013679	-1.49699	2.896873
H	-0.04193	-1.30065	2.996719
C	3.988834	2.750162	-1.68481
H	4.322422	2.861143	-2.70501
C	3.030223	-2.60758	2.799998
H	3.769243	-3.39302	2.766005
C	3.290679	-1.20465	2.781951
H	4.268416	-0.74803	2.746113
C	2.048292	-0.51958	2.842884
H	1.906522	0.548237	2.884692
C	-2.93849	2.889283	-0.45692
H	-2.62507	2.833884	-1.48689
C	-2.38901	3.735577	0.540689
H	-1.57689	4.432594	0.399734
C	-3.10197	3.517794	1.757151
H	-2.91932	4.011204	2.699381
C	-4.08666	2.530075	1.506984
H	-4.7916	2.144616	2.228225
C	-3.99061	2.139608	0.140925
H	-4.62222	1.425105	-0.36119
C	-4.32588	-2.30836	-1.16654
H	-5.13291	-1.90153	-1.75727
C	-4.02223	-1.96447	0.181871
H	-4.56789	-1.27175	0.802002
C	-2.89258	-2.73206	0.581176
H	-2.43918	-2.71717	1.559042
C	-2.49938	-3.54069	-0.5189
H	-1.66949	-4.23089	-0.53531
C	-3.39224	-3.28342	-1.59932
H	-3.3558	-3.74077	-2.57606
C	1.625885	-2.781	2.872112
H	1.107418	-3.7278	2.896883

$[\{(CO)_2CpMn\}_2B\{Cu(IMe)\}]$

Mn	1.127906	-1.88531	-0.23013
Mn	1.127495	1.885727	0.229984
B	1.141249	0.000241	-0.00011

H	-4.59719	-1.17757	-0.67654
C	-4.47434	-1.97156	1.409083
H	-5.18437	-1.36232	1.947586
C	-3.70925	-3.03292	1.959792
H	-3.72089	-3.359	2.988202
C	-2.91138	-3.58064	0.911822
H	-2.20768	-4.39316	1.008701
Au	0.00588	1.44776	0.01655
O	4.655573	1.750254	-0.60581
C	3.741166	1.642245	0.089955
O	1.601196	4.21902	0.340724
Mn	-2.37037	1.480816	-1.22995
C	1.809999	3.11593	0.618331
Mn	2.383215	1.50553	1.216435
C	-1.02391	1.979399	-2.31453
O	-0.28228	2.345067	-3.12062
O	-2.70728	-0.87624	-2.92316
C	-2.56019	0.040779	-2.23302
C	1.37866	1.01803	3.119153
H	0.317752	0.887456	3.255384
C	2.093137	2.236751	3.264713
H	1.660359	3.195893	3.505823
C	3.470573	1.981464	3.0266
H	4.267941	2.7073	3.063138
C	3.609397	0.593654	2.730322
H	4.52967	0.077155	2.50838
C	2.317316	-0.00041	2.782948
H	2.091509	-1.04613	2.651352
C	-3.19388	2.865551	0.280998
H	-2.75303	3.039137	1.248903
C	-4.16033	1.868473	-0.02411
H	-4.59721	1.177519	0.676559
C	-4.4744	1.971475	-1.40908
H	-5.18441	1.362204	-1.94758
C	-3.70934	3.032846	-1.95981
H	-3.72099	3.358911	-2.98823
C	-2.91148	3.580602	-0.91186
H	-2.2078	4.393141	-1.00876

$[\{(CO)_2CpMn\}_2B\{Au(IMe)\}]$

Au	-1.0216	-0.42012	0.006794
Mn	1.61275	1.963204	0.041152
Mn	1.255705	-1.8104	-0.09095

O	-1.15163	-2.43392	-2.02446
C	-0.30261	-2.123	-1.29092
O	2.812914	-1.185	-2.52675
C	2.134831	-1.45908	-1.6262
O	2.816413	1.185553	2.523748
C	2.136765	1.459555	1.624356
O	-1.15009	2.431328	2.027659
C	-0.30167	2.121779	1.292862
C	1.642718	-2.09625	1.878994
H	1.695104	-1.28675	2.589586
C	0.508708	-2.91012	1.607791
H	-0.4648	-2.81965	2.06425
C	0.890728	-3.89026	0.643679
H	0.248705	-4.6515	0.226858
C	2.250945	-3.67995	0.317348
H	2.830262	-4.25342	-0.38999
C	2.720342	-2.56821	1.079815
H	3.719313	-2.16054	1.063312
C	0.506231	2.90847	-1.60849
C	1.642383	2.097326	-1.87907
C	2.718532	2.572224	-1.07969
C	2.246154	3.683067	-0.31773
C	0.885499	3.889817	-0.64455
H	-0.46681	2.815583	-2.06547
H	1.696973	1.287726	-2.58937
H	3.718491	2.166998	-1.06264
H	2.823794	4.25822	0.389613
H	0.241446	4.649577	-0.22816
Cu	-0.86379	-0.00043	-0.00027
C	-2.83581	-0.0004	0.000157
N	-3.67484	0.068857	-1.06994
C	-5.00248	-0.04228	0.67672
C	-5.00289	0.041707	-0.67512
H	-5.81761	-0.08232	1.379022
H	-5.81843	0.081777	-1.37693
N	-3.67422	-0.06958	1.070756
C	-3.24295	0.119131	-2.46325
H	-2.17424	0.317918	-2.48812
H	-3.43078	-0.83513	-2.95737
H	-3.77203	0.919169	-2.98429
C	-3.24147	-0.1202	2.463815
H	-2.17345	-0.32282	2.488092
H	-3.42555	0.835079	2.95737

B	1.169991	0.158705	-0.02803
O	0.390114	2.095362	2.698847
C	0.884178	2.040948	1.645443
O	4.186181	1.062852	1.102689
C	3.163408	1.425836	0.692276
O	2.982274	-1.0383	-2.32246
C	2.283141	-1.31506	-1.44647
O	-0.59244	-3.02513	-2.04243
C	0.084024	-2.49159	-1.26883
C	0.928862	2.216231	-2.01572
H	0.520284	1.422806	-2.62175
C	0.188849	3.097761	-1.17844
H	-0.87974	3.085751	-1.03208
C	1.109003	4.019556	-0.59213
H	0.860546	4.808124	0.101489
C	2.405312	3.704521	-1.06028
H	3.31914	4.207853	-0.78465
C	2.300226	2.581666	-1.93799
H	3.11374	2.099562	-2.4571
C	0.781329	-2.61081	1.906324
C	1.858486	-1.68134	1.998265
C	2.936685	-2.17867	1.218279
C	2.525708	-3.41456	0.633157
C	1.199802	-3.67839	1.067077
H	-0.17457	-2.5261	2.397659
H	1.863904	-0.7743	2.581323
H	3.892013	-1.69418	1.090519
H	3.119834	-4.0408	-0.01376
H	0.603436	-4.53419	0.788973
C	-5.19743	0.475571	-0.40649
C	-4.96554	1.110787	0.767753
C	-3.01779	0.176037	0.109375
N	-3.9961	-0.09379	-0.79466
H	-6.09779	0.380481	-0.98901
H	-5.62634	1.671726	1.406332
N	-3.62748	0.918214	1.068694
C	-2.98307	1.435231	2.275953
H	-3.12669	2.514917	2.336968
H	-3.41148	0.959222	3.160027
H	-1.91816	1.224746	2.230812
C	-3.81445	-0.85133	-2.03181
H	-4.6284	-1.56925	-2.14109
H	-3.80375	-0.17761	-2.89091

H	-3.773	-0.91801	2.985754

$[\{(CO)_2CpMn\}_2B\{Cu(PCy_3)\}]$

Mn	-1.97774	0.720931	0.217721
Mn	1.772668	1.170961	-0.19843
B	-0.10795	1.010621	0.014186
O	-2.31674	-1.78589	1.740306
C	-2.06111	-0.83376	1.116383
O	-1.55696	2.226113	2.700728
C	-1.71887	1.618542	1.726881
O	0.970986	3.004877	-2.34236
C	1.285404	2.268885	-1.50375
O	2.42731	-0.88759	-2.21235
C	2.09008	-0.13204	-1.39394
C	-2.23227	1.623602	-1.75264
H	-1.44151	2.033821	-2.36036
C	-2.7275	0.290738	-1.80359
H	-2.36653	-0.49557	-2.44833
C	-3.80573	0.189104	-0.87689
H	-4.38743	-0.69858	-0.6784
C	-3.98071	1.450553	-0.25661
H	-4.71511	1.693291	0.495761
C	-3.00325	2.339054	-0.79536
H	-2.87675	3.378229	-0.5327
C	2.594451	0.574895	1.75154
C	1.906476	1.814561	1.883826
C	2.55627	2.763212	1.048682
C	3.650681	2.115533	0.40236
C	3.667994	0.766025	0.834283
H	2.363281	-0.33652	2.280422
H	1.065448	2.011199	2.529814
H	2.273959	3.798552	0.932527
H	4.335962	2.569568	-0.29653
H	4.373453	0.011621	0.519553
Cu	0.117137	-0.98381	-0.0462
P	0.387492	-3.2545	-0.03784
C	-0.9293	-4.18331	-0.93547
H	-0.76944	-5.26279	-0.86782
H	-1.89989	-3.93603	-0.50233
H	-0.93399	-3.88937	-1.98688
C	1.939656	-3.93494	-0.76261
H	1.944133	-5.02786	-0.73272
H	2.043422	-3.5938	-1.79345

H	-2.86902	-1.38639	-1.98125

$[\{(CO)_2CpMn\}_2B\{Au(PCy_3)\}]$

Au	-1.21703	0.320054	0.155464
Mn	1.615402	-1.80285	-0.24082
Mn	0.901955	1.908981	0.166509
B	1.025848	-0.05425	-0.02661
O	-0.39535	-2.32056	-2.31067
C	0.408197	-2.09698	-1.49905
O	3.540097	-0.66427	-2.12846
C	2.771018	-1.12336	-1.39073
O	2.981813	1.225049	2.103917
C	2.143144	1.462167	1.346572
O	-0.79564	2.784202	2.417532
C	-0.18026	2.374519	1.530088
C	1.593268	-2.05549	1.924536
H	1.224395	-1.32238	2.624443
C	0.835205	-3.10376	1.333811
H	-0.21213	-3.30016	1.500924
C	1.71564	-3.87667	0.515454
H	1.441235	-4.73717	-0.07513
C	3.003302	-3.29869	0.593708
H	3.883526	-3.64102	0.071609
C	2.933976	-2.16722	1.462511
H	3.751117	-1.51798	1.73408
C	0.089102	2.831865	-1.66392
C	1.202899	2.004628	-1.99054
C	2.344783	2.520964	-1.32257
C	1.937184	3.666149	-0.57187
C	0.548772	3.856885	-0.79257
H	-0.91834	2.714652	-2.02928
H	1.189767	1.152987	-2.65142
H	3.340877	2.110802	-1.37794
H	2.573835	4.284018	0.041888
H	-0.05891	4.634916	-0.35595
P	-3.32842	-0.76094	-0.09893
C	-3.40324	-2.47203	0.568006
H	-2.64087	-3.07894	0.077484
H	-3.20128	-2.45777	1.640344
H	-4.38681	-2.91596	0.392192
C	-4.7739	0.076534	0.671272
H	-4.61308	0.162397	1.747297
H	-4.87216	1.082999	0.261076

H	2.796436	-3.55761	-0.20116	H	-5.69649	-0.48044	0.486563
C	0.343311	-4.01269	1.641835	C	-3.80063	-0.95913	-1.86279
H	-0.59373	-3.74163	2.130544	H	-3.88465	0.02415	-2.32905
H	0.42582	-5.10128	1.584364	H	-3.0166	-1.51812	-2.37689
H	1.16946	-3.6256	2.241679	H	-4.7533	-1.48686	-1.95935

References.

1. H. Braunschweig, M. Burzler, R. D. Dewhurst and K. Radacki, *Angew. Chem. Int. Ed.* 2008, **47**, 5650–5653.

2. Prepared by a variation on the methods in: P. Braunstein, H. Lehner and D. Matt, *Inorg. Synth*. 1990, **27**, 218.

3. H. Braunschweig, K. Kraft, S. Ostreicher, K. Radacki and F. Seeler, *Chem. Eur. J.*, 2010, **16**, 10635.

4. W. L. F. Armarego and C. L. L. Chai, *Purification of Laboratory Chemicals; 6 ed*., Elsevier: Oxford, **2009**.

5. G. M. Sheldrick, *Acta Crystallogr., Sect. A Found. Crystallogr.* 2008, **A64**, 112.

6. C. B. Huebschle, G. M. Sheldrick, B. Dittrich, *J. Appl. Crystallogr*. 2011, **44**, 1281.

7. C. Cason, et al.; Persistence of Vision Pty. Ltd., 2009; http://www.povray.org/download/.

8. (a) G. te Velde, F. M. Bickelhaupt, E. J. Baerends, C. Fonseca Guerra, S. J. A. van Gisbergen, J. G. Snijders, and T. Ziegler. *J. Comp. Chem*., 2001, **22**, 931; (b) Amsterdam Density Functional, Theoretical Chemistry, Vrije Universiteit, Amsterdam, The Netherlands, http://www.scm.com.

9. (a) N. C. Handy and A. J. Cohen, *Mol. Phys.,* 2001, **99**, 403; (b) D. P. Chong, *Mol. Phys*., 2005, **103**, 749; (c) D. P. Chong, E. van Lenthe, S. J. A. van Gisbergen and E. J. Baerends, *J.*

Comp. Chem., 25, **2004**, 1030; (e) E. van Lenthe and E. J. Baerends. *J. Comp. Chem*., 2003, **24**, 1142; (f) R. C. Raffenetti, *J. Chem. Phys*., 1973, **59**, 5936.

10. (a) E. van Lenthe, E. J. Baerends, and J. G. Snijders, *J. Chem. Phys*., 1993, **99**, 4597; (b) E. van Lenthe, E. J. Baerends and J. G. Snijders, *J. Chem. Phys*., 1994, **101**, 9783; (c) E. van Lenthe, A. E. Ehlers and E. J. Baerends. *J. Chem. Phys.*, 1999, **110**, 8943; (d) R. Bouten, E. J. Baerends, E. van Lenthe, L. Visscher, G. Schreckenbach and T. Ziegler, *J. Phys. Chem. A*, 2000, **104**, 5600.

11. (a) I. Mayer, *Chem. Phys. Lett*., 1983, **97**, 270; (b) E. P. Fowe, B. Therrien, G. Süss-Fink, and C. Daul, *Inorg. Chem*., 2008, **47**, 42.

12. (a) F. L. Hirshfeld. *Theo. Chim. Acta*, 1993, **44**, 129; (b) K. B. Wiberg and P. R. Rablen. *J. Comp. Chem*., 1993, **14**, 1504.

13. S. Grimme, S. Ehrlich, and L. Goerigk, *J. Comp. Chem*., 2011, **32**, 1456.

14. Jmol: an open-source Java viewer for chemical structures in 3D. http://www.jmol.org/.

15. Gaussian 09, Revision D.01, M. J. Frisch, G. W. Trucks, H. B. Schlegel, G. E. Scuseria, M. A. Robb, J. R. Cheeseman, G. Scalmani, V. Barone, B. Mennucci, G. A. Petersson, H. Nakatsuji, M. Caricato, X. Li, H. P. Hratchian, A. F. Izmaylov, J. Bloino, G. Zheng, J. L. Sonnenberg, M. Hada, M. Ehara, K. Toyota, R. Fukuda, J. Hasegawa, M. Ishida, T. Nakajima, Y. Honda, O. Kitao, H. Nakai, T. Vreven, J. A. Montgomery, Jr., J. E. Peralta, F. Ogliaro, M. Bearpark, J. J. Heyd, E. Brothers, K. N. Kudin, V. N. Staroverov, R. Kobayashi, J. Normand, K. Raghavachari, A. Rendell, J. C. Burant, S. S. Iyengar, J. Tomasi, M. Cossi, N. Rega, J. M. Millam, M. Klene, J. E. Knox, J. B. Cross, V. Bakken, C. Adamo, J. Jaramillo, R. Gomperts, R. E. Stratmann, O. Yazyev, A. J. Austin, R. Cammi, C. Pomelli, J. W. Ochterski, R. L. Martin, K. Morokuma, V. G.

Zakrzewski, G. A. Voth, P. Salvador, J. J. Dannenberg, S. Dapprich, A. D. Daniels, Ö. Farkas, J. B. Foresman, J. V. Ortiz, J. Cioslowski, and D. J. Fox, Gaussian, Inc., Wallingford CT, 2009.
